# Remdesivir inhibits endothelial activation and atherosclerosis by coupling TAL1 to TRAF6

**DOI:** 10.1186/s12967-025-06673-2

**Published:** 2025-07-01

**Authors:** Hanning Zhang, Ruru Li, Qianqian Huo, Li Li, Min Li, Shunxin Hu, Changjie Ren, Zongyin Wu, Chenghu Zhang

**Affiliations:** 1 Shandong First Medical University, Jinan, China; 2 Department of Cardiology, Jining First People’s Hospital, Shandong First Medical University, No. 6 Jiankang Road, Jining, 272000 Shandong China; 3 Jining Key Laboratory of Metabolic Cardiovascular Diseases, Jining, China

## Abstract

**Background:**

Atherosclerosis is characterized by complex pathological processes, including endothelial dysfunction and inflammation. The underlying pathogenic mechanisms have been well elucidated; however, effective treatments are yet to be validated. Our study explored the novel application of a recognized antiviral agent, remdesivir, focusing on its impact on endothelial activation and atherosclerosis.

**Methods:**

Pharmacological treatment with remdesivir significantly reduced atherosclerotic lesions in the total aorta and decreased VCAM-1 expression in aortic roots of ApoE−/− mice. Remdesivir notably attenuated ox-LDL-induced endothelial cell (EC) activation, monocyte adhesion, and ROS production. In HUVECs, TAL1 interference via siRNA significantly increased TRAF6 protein levels, which was reversed by remdesivir. Remdesivir also reduced both total and K63-linked ubiquitination of TRAF6 in HUVECs. Immunoprecipitation assays revealed diminished co-localization of the two proteins under ox-LDL treatment, but this effect was reversed by remdesivir. Importantly, ectopic adeno-associated virus (AAV)-mediated overexpression of TAL1 reduced atherosclerotic lesions and VCAM-1 expression in the aorta of ApoE−/− mice.

**Results:**

In ApoE^–/–^ mice fed with a Western diet, remdesivir greatly attenuated atherosclerosis progression. At the cellular level, remdesivir suppressed oxidative stress, THP-1 adhesion, vascular cell adhesion molecule 1, and intercellular adhesion molecule 1 via oxidized low-density lipoprotein in human umbilical vein endothelial cells. T-cell acute lymphoblastic leukemia 1 (TAL1) functions by interacting with the ubiquitin E3 ligase TNF receptor-associated factor 6 (TRAF6) to ubiquitinate TRAF6, inhibiting nuclear factor kappa B activation. Remdesivir also restored the TAL1-TRAF6 interaction and decreased endothelial activation. Endothelial-specific TAL1 over-expression in ApoE^–/–^ mice significantly reduced aortic plaque formation.

**Conclusions:**

Remdesivir impedes atherosclerosis progression by re-establishing the interaction between TAL1 and TRAF6, diminishing endothelial activation. These findings offer an innovative therapeutic approach for atherosclerosis.

**Supplementary Information:**

The online version contains supplementary material available at 10.1186/s12967-025-06673-2.

## Introduction

Atherosclerosis is a major contributor to cardiovascular disease and stroke [1]. In the early stages, increase in the plasma low-density lipoprotein (LDL) levels is generally associatedwith atherosclerosis. In pathological conditions, the LDL is modified into oxidized low-density lipoprotein (ox-LDL) [2]. Furthermore, ox-LDL triggers endothelial cell activation, producing reactive oxygen species (ROS) and causing the nuclear translocation of nuclear factor kappa B (NF-κB). This increases the expression of vascular cell adhesion molecule 1 (VCAM-1) and intercellular adhesion molecule 1 (ICAM-1) [3–6]. TNF receptor-associated factor 6 (TRAF6), a crucial component of the NF-κB signaling pathway, significantly influences the inflammatory process. It forms K63 polyubiquitin chains via interacting with Ubc13 and Uev1A, activating the transformation of growth factor β-activated kinase 1 (TAK1) and inhibiting kappa B kinase (IKK) and NF-κB [7, 8]. TRAF6 is unique among E3 ubiquitin ligases due to its ability to undergo autoubiquitination, dependent on its really interesting new gene (RING) finger domain [9]. While the K63 polyubiquitin chains of TRAF6 mediate the NF-κB pathway, its K48 polyubiquitin chains are degraded by the 26S proteasome [9–11]. The central role of K63 ubiquitination in the pathway highlights a potential target for therapeutic intervention, suggesting that inhibiting this process could be a strategic approach for preventing and treating inflammatory diseases.

Lectin-like oxidized low-density lipoprotein (LDL) receptor-1 (LOX-1) was initially identified as the major receptor for oxidized LDL (ox-LDL) in endothelial cells. Upon interaction with ox-LDL, LOX-1 triggers endothelial dysfunction marked by reactive oxygen species-mediated activation of NF-κB signaling pathways, leading to upregulated adhesion molecule expression and programmed cell death in endothelial tissue [12]. Emerging data position LOX-1 as a multifaceted contributor to atherosclerotic progression, influencing various pathological mechanisms underlying plaque formation. Cellular studies reveal that LOX-1 expression becomes elevated under pro-inflammatory conditions, oxidative challenges, mechanical stress, and paradoxically, in response to its primary ligand ox-LDL. In disease models, heightened LOX-1 levels correlate with atherogenic risk factors such as metabolic dysregulation, vascular hypertension, and diabetic complications, with notable accumulation observed in atherosclerotic plaques and renal pathological formations [13]. Current investigative efforts focus on pharmacological modulation of this receptor-ligand interaction, with ongoing clinical research exploring LOX-1 inhibition as a potential therapeutic strategy for managing ischemic heart disease and related cardiovascular pathologies [14]. These translational studies aim to establish proof-of-concept for targeting this pathway in vascular disease management.

Remdesivir (RDV), a prodrug of adenosine nucleotide analogs, is known for its broad-spectrum antiviral activity achieved by inhibiting RNA-dependent RNA polymerase [15, 16]. Initially, remdesivir was recognized for its effectiveness in preventing Ebola, mitigating acute kidney, suppressing severe acute respiratory syndrome coronavirus 2 (SARS-CoV-2) replication and reducing inflammation during the coronavirus disease (COVID-19) pandemic [17–19]. More importantly, remdesivir exhibited potential therapeutic effects on non-viral inflammation-related conditions beyond its antiviral properties. It also alleviates non-alcoholic fatty liver disease induced by a high-fat diet by inhibiting the stimulator of interferon genes [18]. Whether remdesivir can participate in the process of atherosclerosis has not been reported.

T-cell acute lymphoblastic leukemia 1 (TAL1) or stem cell leukemia (SCL), is a crucial transcription factor in the hematopoietic system and the most common oncogenes in T-cell acute lymphoblastic leukemia [20]. Its activity relies on its function within a core protein complex comprising TAL1, E-protein, LIM domain only 1 or 2 (LMO1/2), and LIM domain-binding protein 1 [21]. TAL1 and LMO2 are essential in angiogenic remodeling, inducing blood vessel cells to differentiate into endothelial cells without erythroid or myeloid hematopoietic inducers [22]. TAL1 and LMO2 are key factors that directly transform adult skin fibroblasts into endothelial cells [23]. TAL1 expression in monocytes increased significantly under SARS-CoV-2 regulation, which was effectively mitigated by remdesivir [24]. However, the precise mechanism by which TAL1 impacts endothelial cell activation is still undefined. Whether remdesivir could ameliorate atherosclerosis through TRAF6 ubiquitination regulated by TAL1 remains unclear. Our study found that the interaction between TAL1 and TRAF6 increased by remdesivir, reducing endothelial activation. This study demonstrated the potential role of remdesivir against endothelial dysfunction and atherosclerosis.

## Results

### Remdesivir reduced endothelial cell activation and atherosclerosis

**Fig. 1:**
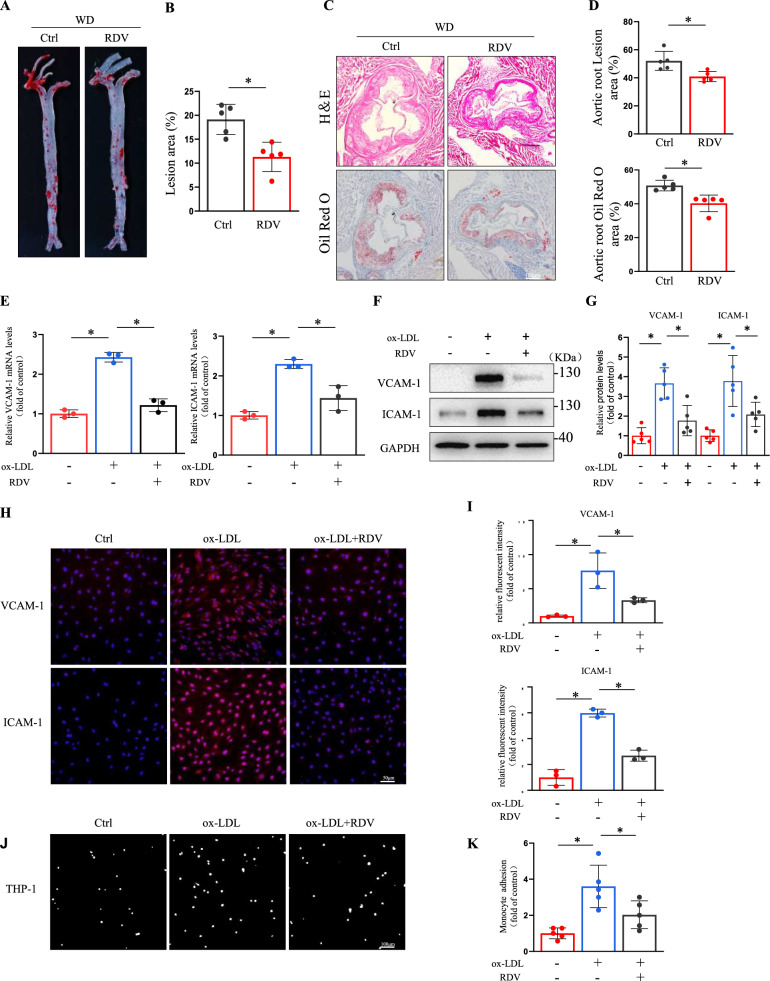
Remdesivir reduced endothelial cell activation and atherosclerosis. Male ApoE^–/–^ mice were treated with remdesivir (15 mg/kg) or DMSO every two days and fed a Western diet for four weeks. **A** Representative en face preparations of whole aortas representing atherosclerosis, visualized by Oil Red O staining. **B** Quantification of lesion area in the whole aorta (n = 5 for each group, unpaired, two-tailed Student’s t-test and *P < 0.05). **C, D** Hematoxylin and eosin and Oil Red O staining of the aortic root, scale bar = 100 μm (n = 5 for each group, unpaired, two-tailed Student’s t-test and *P < 0.05). **E** Treatment of HUVECs with ox-LDL and remdesivir for 24 h, followed by RT-PCR detection of VCAM-1 and ICAM-1 RNA levels (n = 3 for each group, one-way ANOVA with Bonferroni multiple comparison post-hoc test and *P < 0.05). **F, G** Representative Western blots of VCAM-1 and ICAM-1 (n = 5 for each group, one-way ANOVA with Bonferroni multiple comparisons post-hoc tests and *P < 0.05). **H, I** Immunofluorescence staining of VCAM-1 and ICAM-1, red fluorescence indicates VCAM-1 and ICAM-1, and blue fluorescence represents DAPI, scale bar = 50 μm. Quantification of relative fluorescence intensity in three random fields (n = 3 for each group, one-way ANOVA with Bonferroni multiple comparisons post-hoc tests and *P < 0.05). **J, K** Treatment of endothelial cells with ox-LDL and remdesivir for 24 h, followed by a cell adhesion assay with fluorescently labeled THP-1, scale bar = 100 µm. The number of adhesive cells was quantified from five random fields with a 10 × objective in each experiment and was normalized to control. Scale bar = 100 µm (n = 5 for each group, one-way ANOVA with Bonferroni multiple comparisons post-hoc tests and *P < 0.05)

To investigate the effect of remdesivir on atherosclerosis, male and female ApoE^–/–^ mice were treated with Remdesivir (15 mg/kg) or dimethyl sulfoxide (DMSO) and fed a Western diet for four weeks [25]. Remdesivir significantly reduced atherosclerotic lesions in the aortic arch and thoracic aorta (Fig. 1A, B, Supplemental Fig. 1A-B). Oil Red O and hematoxylin–eosin (H&E) staining revealed that remdesivir reduced lesion area and lipid deposition in aortic roots (Fig. 1C, D, Supplemental Fig. 1C-D), indicating that remdesivir might play an essential role in atherosclerosis. Given the critical role of endothelial adhesion molecules such as VCAM-1 and ICAM-1 in mediating leukocyte recruitment during atherogenesis, we analyzed VCAM-1 expression levels in aortic roots of mice. Atherosclerosis was reduced in remdesivir-treated mice along with decreased VCAM-1 expression in aortic roots of ApoE −/− mice (Supplemental Fig. 1E-F, Supplemental Fig. 2A-B), which indicates potential anti-inflammatory effects of remdesivir. In addition, remdesivir had few effects on plasma total triglyceride, total cholesterol, and LDL cholesterol levels, although we observed a mild elevation in transaminases (ALT and AST) associated with its administration in mice(Supplemental Fig. 3A-B). Because endothelial cell activation is critical in initiating atherosclerosis, ox-LDL can induced oxidative stress and inflammatory response in endothelial cells, [26] so we treated human umbilical vein endothelial cells (HUVECs) with ox-LDL (100 μg/ml) or Remdesivir. At doses of 1 to 20 μM, remdesivir did not have detectable cytotoxicity in HUVECs, we next treated HUVECs with remdesivir (10 μM) (Supplemental Fig. 4A-E). We found that remdesivir significantly reversed ox-LDL-induced VCAM-1 and ICAM-1 activation in HUVECs and human aortic endothelial cells (HAECs) (Fig. 1E–I, Supplemental Fig. 5A-C). Notably, remdesivir showed no significant effect on the expression of macrophage-derived inflammatory cytokines(Supplemental Fig. 6A-B). To investigate the deposited lipid effect on the ECs under oxidative stress, HUVECs exposed to ox-LDL were stained with Oil Red O. As shown in Supplemental Fig. 7A-B, the results demonstrated that compared with the control group, lipid droplets in HUVECs treated with ox-LDL for 24 h were significantly increased, which indicates that HUVECs were lipid-loaded. However, when HUVECs were treated with remdesivir, lipid deposition in the cells was significantly decreased compared with ox-LDL group. The present data indicated that prolonged exposure to ox-LDL led to elevated lipid accumulation in the ECs. Furthermore, remdesivir mitigated the number of THP-1 cells adhering to HUVECs induced by ox-LDL (Fig. 1J, K). Our results demonstrated that remdesivir effectively decreased ox-LDL-induced endothelial activation and atherosclerosis.

### Remdesivir inhibited lectin-like oxidized low-density lipoprotein receptor-1 (LOX-1) production in endothelial cells

**Fig. 2:**
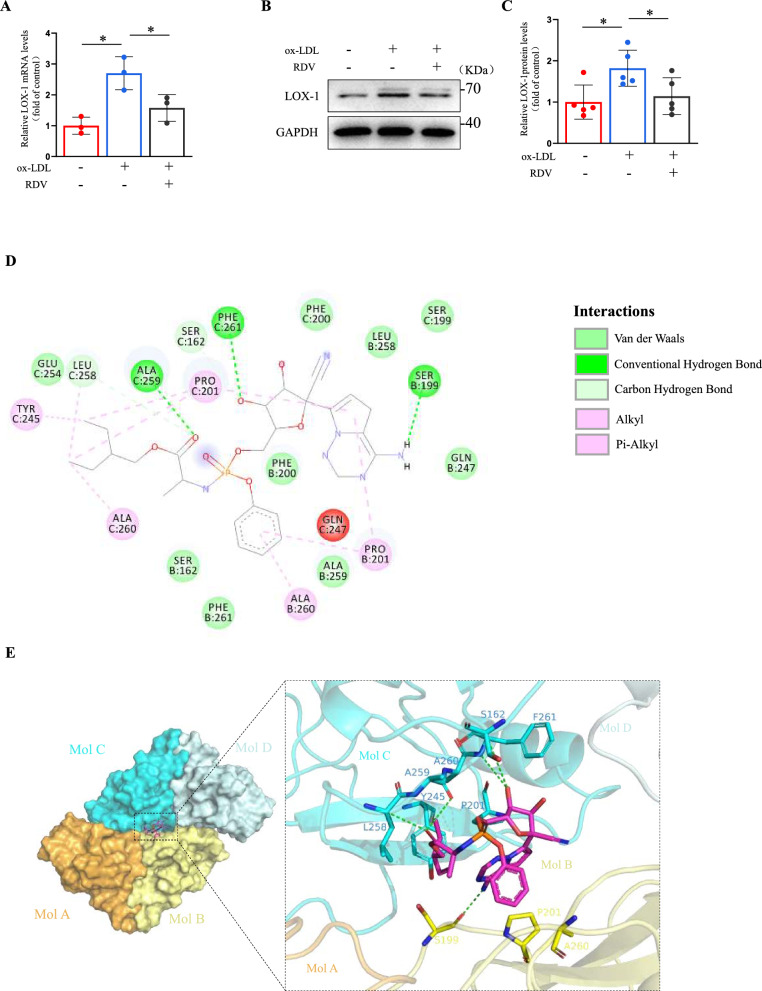
Remdesivir inhibited the production of LOX-1 in endothelial cells. **A** Treatment of HUVECs with ox-LDL and remdesivir for 24 h, followed by RT-PCR detection of LOX-1 mRNA levels (n = 3 for each group, one-way ANOVA with Bonferroni multiple comparison post-hoc test and *P < 0.05). **B, C** Representative Western blots of LOX-1 (n = 5 for each group, one-way ANOVA with Bonferroni multiple comparison post-hoc test and *P < 0.05). **D** Binding sites of LOX-1 and remdesivir predicted by AutoDock4.2.6. The (**D**) 2D and (**E**) 3D binding modes of remdesivir and LOX-1 tetramer. Molecules A, B, C, and D correspond to distinct monomeric conformations of the LOX-1 receptor. The remdesivir is depicted as magenta sticks. The surrounding residues in the binding pocket are presented as cyan and yellow sticks. The hydrogen bonds are depicted as green dashed lines

LOX-1 is the primary ox-LDL receptor in endothelial cells, contributing to endothelial dysfunction [12]. So we next analyzed the mRNA and protein levels of LOX-1 to explore the effect of remdesivir on LOX-1. LOX-1 expression in cells exposed to ox-LDL was effectively inhibited by remdesivir in HUVECs and HAECs (Fig. 2A–C, Supplemental Fig. 5 A, 5D). However, we observed that while ox-LDL upregulated CD36 expression, remdesivir showed no inhibitory effect on CD36. Furthermore, scavenger receptor A1 (SR-A1) levels remained unaltered, suggesting that RDV’s anti-inflammatory activity may be specifically mediated through LOX-1 regulation (Supplemental Fig. 8A-C). LOX-1 is a transmembrane protein comprising four domains, and its hydrophobic tunnel is essential for the recognition and binding of ox-LDL [27]. Molecular docking exposed that remdesivir formed a suitable steric complementarity with the binding site of two dimers of LOX-1. The remdesivir formed four hydrogen bonds with Leu258, Ala259, Phe261 of molecule C and Ser199 of molecule B. Remdesivir formed hydrophobic interactions with LOX-1 tetramer involving residues Leu258, Tyr245, Pro201, Ala260 of molecule C and Pro201 and Ala260 of molecule B. Van der Waals interactions were also formed among remdesivir and LOX-1 (Fig. 2D, E). This suggested that remdesivir may exert anti-atherosclerotic effects through the LOX-1 signaling pathway.

### Remdesivir negatively regulated TAL1 in endothelial cells

**Fig. 3:**
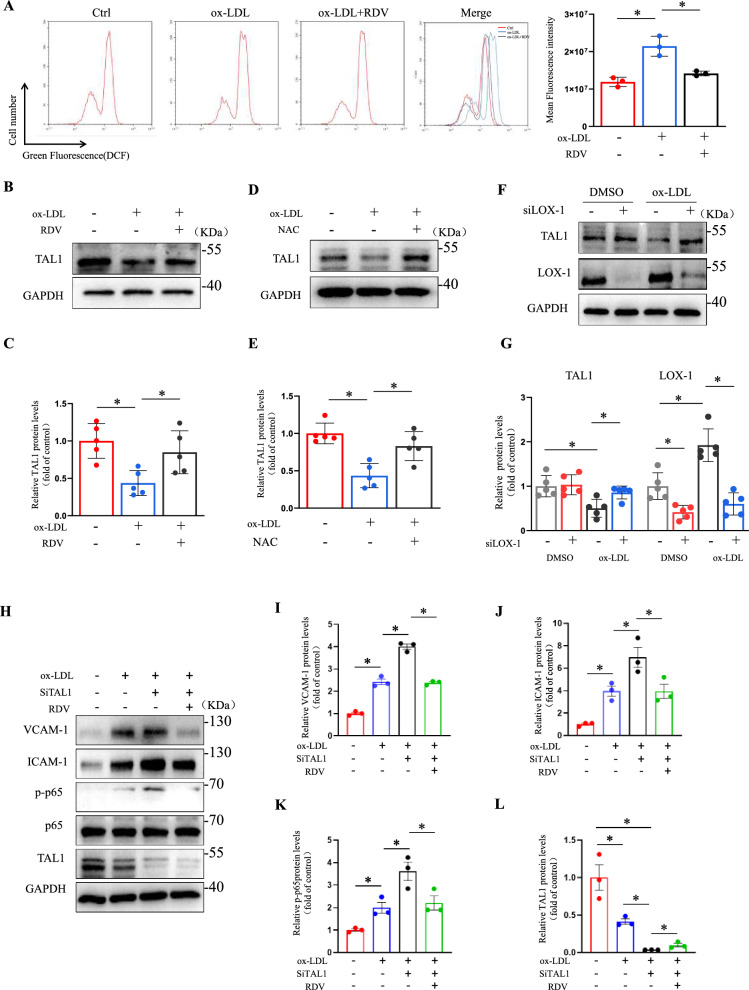
Remdesivir negatively regulated TAL1 in endothelial cells. **A** Treatment of endothelial cells with ox-LDL and remdesivir for 24 h, ROS levels were detected using the DCFH-DA assay kit (n = 3 for each group, one-way ANOVA with Bonferroni multiple comparison post-hoc test, and *P < 0.05). **B, C** Treatment of HUVECs with ox-LDL and remdesivir for 24 h, representative Western blots of TAL1 (n = 5 for each group, one-way ANOVA with Bonferroni multiple comparison post-hoc test and *P < 0.05). **D, E** Treatment of HUVECs with ox-LDL and NAC for 24 h, representative Western blots of TAL1 (n = 5 for each group, one-way ANOVA with Bonferroni multiple comparison post-hoc test and *P < 0.05). **F, G** HUVECs transfected with siLOX-1 or DMSO for 24 h, treated with ox-LDL for another 24 h, quantification analysis of TAL1 and LOX-1 by Western blotting (n = 5 for each group, two-way ANOVA with Bonferroni multiple comparison post-hoc test and *P < 0.05). **H–L** HUVECs transfected with siTAL1 or DMSO for 24 h, treated with ox-LDL and remdesivir for another 24 h, quantification analysis of VCAM-1, ICAM-1, p-p65, p65 and TAL1 by Western blotting (n = 3 for each group, two-way ANOVA with Bonferroni multiple comparison post-hoc test and *P < 0.05)

Reportedly, the binding of ox-LDL to LOX-1 promoted ROS production [28]. We investigated whether remdesivir mediates endothelial cell activation by influencing ROS production. Flow cytometry was used to detect ROS. HUVECs exposed to ox-LDL exhibited increased ROS production, which was counteracted by remdesivir (Fig. 3A). Because remdesivir can reduced TAL1 expression, which was significantly upregulated by SARS-CoV-2 in monocytes, [24] suggesting a potential regulatory effect of remdesivir on TAL1. we next explored whether TAL1 is affected by remdesivir in endothelial cells. Endothelial cells stimulated with ox-LDL for 24 h significantly reduced TAL1 expression, which was restored by remdesivir (Fig. 3B, C). Furthermore, ROS inhibitor N-acetylcysteine (NAC) significantly reversed the decrease in TAL1, mediated by ox-LDL, suggesting that TAL1 is regulated by ROS (Fig. 3D, E). LOX-1 silencing also reduced the effect of ox-LDL on TAL1 in endothelial cells (Fig. 3F, G, Supplemental Fig. 11 A). To determine whether remdesivir directly modulates TAL1 activity, we performed TAL1 knockdown in endothelial cells under ox-LDL stimulation. Silencing TAL1 significantly elevated protein levels of VCAM-1 and ICAM-1, accompanied by increased phosphorylation of p65 (p-p65) in the NF-κB pathway. Notably, RDV treatment reversed these ox-LDL-induced pro-inflammatory effects in TAL1-silenced cells (Fig. 3H–L, Supplemental Fig. 11B). This indicated that remdesivir could regulate TAL1-mediated endothelial activation.

### Remdesivir modulated ubiquitination and activation of TRAF6 in endothelial cells

**Fig. 4:**
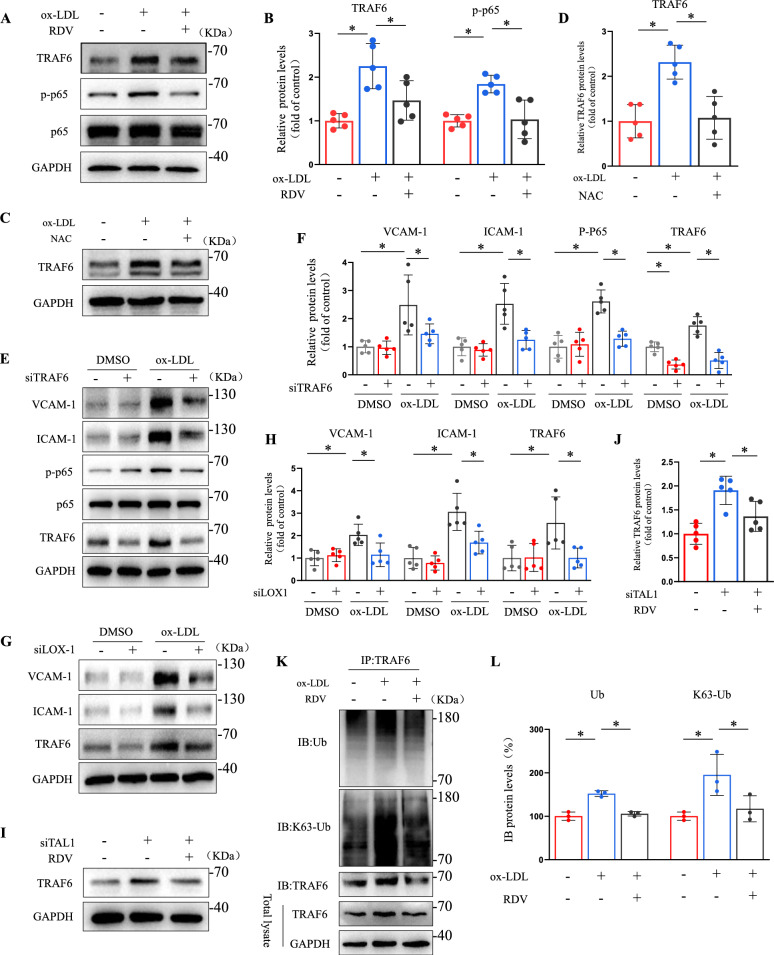
Remdesivir modulated ubiquitination and activation of TRAF6. **A, B** Treatment of HUVECs with ox-LDL and remdesivir for 24 h, representative Western blots of TRAF6, p-p65, and p65 (n = 5 for each group, one-way ANOVA with Bonferroni multiple comparison post-hoc test and *P < 0.05). **C, D** Treatment of HUVECs with ox-LDL and NAC for 24 h, representative Western blots of TRAF6 (n = 5 for each group, one-way ANOVA with Bonferroni multiple comparison post-hoc test and *P < 0.05). **E, F** HUVECs were transfected with siTRAF6 for 24 h and treated with ox-LDL or DMSO for another 24 h. Quantification analysis of VCAM-1, ICAM-1, p-p65, p65 and TRAF6 by Western blotting (n = 5 for each group, two-way ANOVA with Bonferroni multiple comparisons post-hoc tests and *P < 0.05). **G, H** HUVECs were transfected with siLOX-1 or DMSO for 24 h and treated with ox-LDL for another 24 h. Quantification analysis of VCAM-1, ICAM-1, and TRAF6 using Western blotting (n = 5 for each group, two-way ANOVA with Bonferroni multiple comparisons post-hoc tests and *P < 0.05). **I, J** HUVECs transfected with siTAL1 or DMSO for 24 h, treated with remdesivir for another 24 h, and quantification analysis of TRAF6 by Western blotting (n = 5 for each group, one-way ANOVA with Bonferroni multiple comparison post-hoc test and *P < 0.05). **K, L** Treatment of HUVECs with ox-LDL and remdesivir for 24 h, TRAF6 immunoprecipitation followed by representative blots of TRAF6, Ub and K63-Ub (n = 3 for each group, one-way ANOVA with Bonferroni multiple comparison post-hoc test and *P < 0.05)

E3 ubiquitin ligase TRAF6 regulates endothelial cell stimulation through ubiquitination in its RING domain, TAK1 kinase, and NF-κB signaling, while ROS mediates phosphorylation of p65 by regulating ubiquitination of TRAF6 [29, 30]. We examined whether remdesivir regulates TRAF6 and NF-κB signaling pathways in endothelial cells. An increase in TRAF6 and p-p65 protein levels in endothelial cells upon ox-LDL stimulation was effectively reduced by remdesivir (Fig. 4A, B). Moreover, NAC significantly reversed the increase of TRAF6 mediated by ox-LDL, suggesting that TRAF6 is also regulated by ROS (Fig. 4C, D). TRAF6 depletion in HUVECs significantly reduced VCAM-1, ICAM-1, and p-p65 expression induced by ox-LDL (Fig. 4E, F, Supplemental Fig. 11 C). LOX-1 silencing reduced TRAF6 protein expression, indicating a LOX-1-dependent mechanism for ox-LDL-induced TRAF6 activation (Fig. 4G, H). Furthermore, we knocked down TAL1 in endothelial cells while providing remdesivir stimulation. TAL1 knockdown significantly increased TRAF6 protein levels, which was reversed by remdesivir (Fig. 4I), suggesting that remdesivir regulated TRAF6 expression via TAL1. Because polyubiquitination of the K63- link of TRAF6 is essential for TAK1 kinase activation and NF-κB signaling [31]. we found that ox-LDL significantly increased TRAF6 ubiquitination; however, remdesivir decreased the total and K63-linked ubiquitination of TRAF6 in HUVECs (Fig. 4K, L). These results demonstrated that remdesivir modulated TRAF6 ubiquitination and activation in endothelial cells.

### TAL1 interacts with TRAF6 and inhibits K63 ubiquitination in endothelial cells

**Fig. 5:**
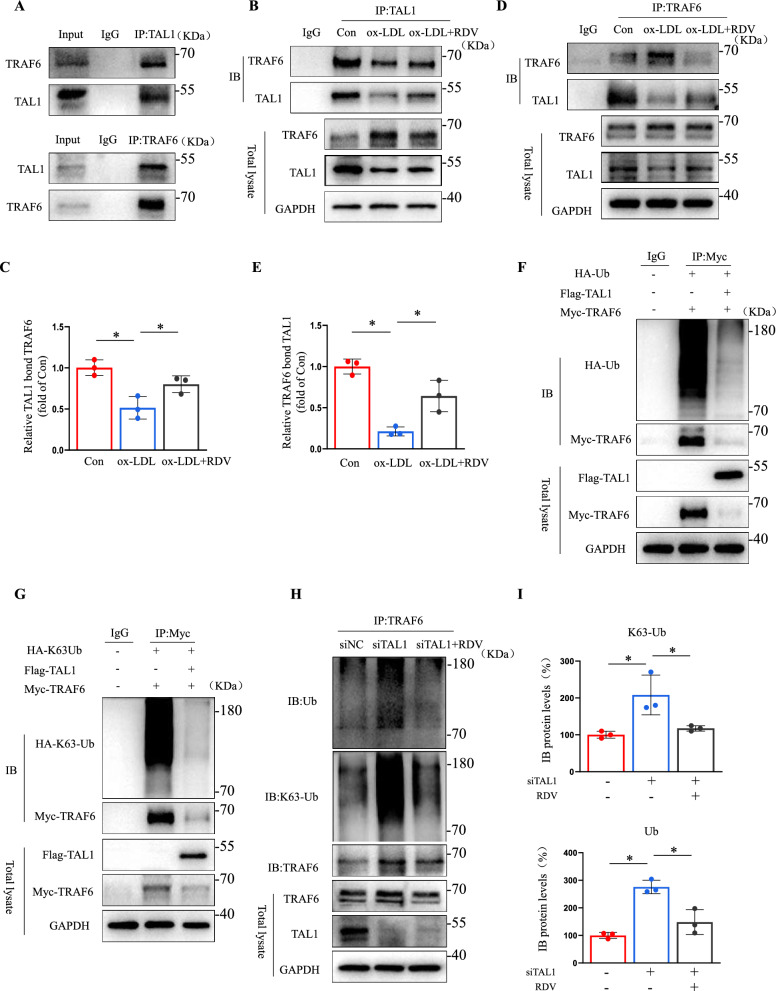
TAL1 interaction with TRAF6 and inhibits K63 ubiquitination. **A** TAL1 and TRAF6 immunoprecipitation followed by representative blots of TAL1 and TRAF6 (n = 3). **B, C** Treatment of HUVECs with ox-LDL and remdesivir for 24 h, TAL1 immunoprecipitation followed by representative blots of TAL1 and TRAF6 (n = 3 for each group, one-way ANOVA with Bonferroni multiple comparison post-hoc test and *P < 0.05). **D, E** Treatment of HUVECs with ox-LDL and remdesivir for 24 h, TRAF6 immunoprecipitation followed by representative blots of TRAF6, and TAL1 (n = 3 for each group, one-way ANOVA with Bonferroni multiple comparison post-hoc test and *P < 0.05). **F, G** HEK293T treated with or without Flag-TAL1 and Myc-TRAF6 co-transfected with HA-Ub or HA-K63 into HEK293T cells for 48 h. Immunoprecipitation with anti-Myc beads followed by representative blots of Myc, HA (n = 3). **H, I** HUVECs transfected with siTAL1 or DMSO for 24 h, treated with remdesivir for another 24 h, TRAF6 immunoprecipitation followed by representative blots of TRAF6, Ub and K63-Ub (n = 3 for each group, one-way ANOVA with Bonferroni multiple comparison post-hoc test and *P < 0.05)

We analyzed the possibility of a direct interaction between TAL1 and TRAF6. Total proteins from ECs were extracted. Immunoprecipitation assays were performed with antibodies recognizing endogenous TAL1 or TRAF6, followed by immunoblotting with the indicated antibodies. The results revealed that TAL1 bound to TRAF6 under static conditions (Fig. 5A). Endothelial cells were treated with ox-LDL for 24 h, and the co-localization of the two proteins was reduced. However, when endothelial cells were treated with ox-LDL and remdesivir, the interaction of TAL1 with TRAF6 significantly increased compared to treatment with ox-LDL alone (Fig. 5B–E and Supplemental Fig. 9A-B). We further explored the possibility of TAL1 on the ubiquitination of TRAF6. We expressed Flag-tagged TAL1 (Flag-TAL1) or HA-tagged Ub (HA-Ub) together with Myc-his-tagged TRAF6 (Myc-TRAF6) in human embryonic kidney 293 T (HEK293T) cells. The association of TAL1 and TRAF6 was assessed by immunoprecipitation of Myc-his-TRAF6, followed by immunoblot analysis of Flag-TAL1 and HA-Ub. TAL1 over-expression significantly reduced K63 ubiquitination and the total ubiquitination levels of TRAF6, suggesting that TAL1 inhibited the TRAF6 activation (Fig. 5F, G). TAL1 deletion in HUVECs increased the total and K63-linked ubiquitination of TRAF6. Remdesivir limited the total and K63-linked ubiquitination of TRAF6 by immunoprecipitation of TRAF6 (Fig. 5H, I). These findings suggested that the interaction between TAL1 and TRAF6 plays a central role in regulating endothelial cell activation.

### Direct interaction between the C-terminal core domain of TAL1 and TRAF6

**Fig. 6:**
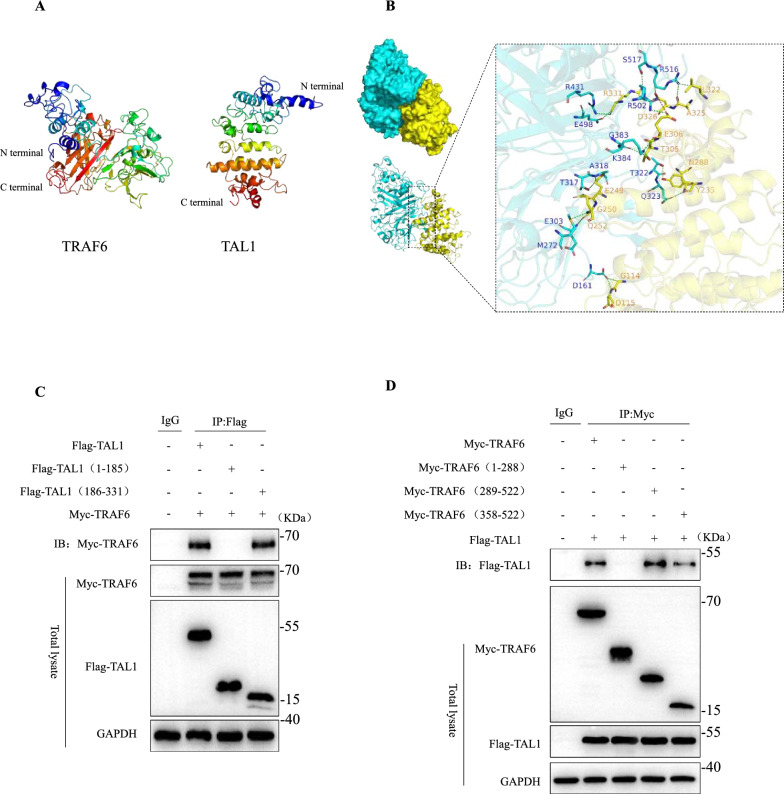
Direct interaction between the C-terminal core domain of TAL1 and TRAF6. **A** The predicted models of TRAF6 (left) and TAL1 (right). **B** The complex model from docking result for TRAF6 and TAL1. The TRAF6 is colored in cyan. The TAL1 is colored in yellow. The contact residues in TRAF6 are presented as cyan sticks, and residues in TAL1 are depicted as yellow sticks. The green dashes represent hydrogen bonds, and the red dashes represent salt bridges. **C, D** After co-transfection of HEK293T cells with the required plasmids for 48 h, immunoprecipitation with anti-Flag or anti-Myc beads, and immunoblotting with specific antibodies

We mapped the binding regions of the TRAF6-TAL1 complex. The predicted models for TRAF6 and TAL1 are displayed in Fig. 6A. The C-scores of TRAF6 and TAL1 models were –1.94 and –2.74, respectively, indicating that the models were in reasonable confidence. The interactions between TRAF6 and TAL1 and the detailed contacts between the two proteins are provided in Fig. 6A and B, TRAF6 also formed 18 hydrogen bonds and one salt bridge with TAL1 (Supplemental Table 1). Van der Waals interactions were observed between TRAF6 and TAL1. These interactions mainly contribute to the binding energy between TRAF6 and TAL1. Flag-tagged full-length (FL) TAL1, Flag-tagged (1-185aa) TAL1, or Flag-tagged (186-331aa) TAL1 were expressed in HEK293T cells with Myc-His-tagged FL TRAF6. Flag-tagged (186-331aa) TAL1 bound to TRAF6 rather than Flag-tagged (1-185aa) TAL1, suggesting that the CTD of TAL1 predominantly contributed to this interaction (Fig. 6C). Flag-tagged FL TAL1 was transiently expressed in HEK293T cells along with Myc-his-tagged FL TRAF6 and its truncated mutants. As presented in Fig. 6D, Flag-TAL1 specifically co-precipitated with Myc-his-TRAF6 and two truncated mutants, except for the Myc-TRAF6 (1–288 aa) mutant. These results suggested that the C-terminal 186–331 region of TAL1 interacted with the C-terminal of TRAF6, consistent with the protein docking.

### Endothelial-specific TAL1 overexpression decreased atherosclerosis

**Fig. 7:**
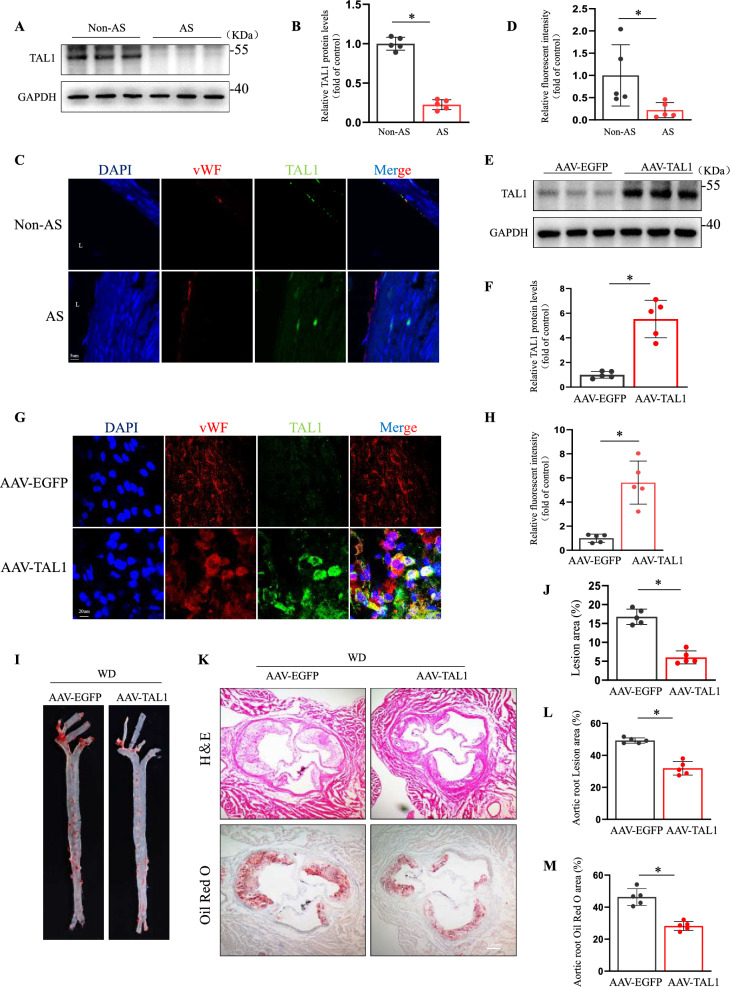
Endothelial-specific over-expression of TAL1 decreased atherosclerosis. **A, B** Human atherosclerotic vessels were divided into atherosclerosis (AS) and Non-AS groups. Western blotting analysis of TAL1 protein levels in vessels. (n = 5 for each group, unpaired, two-tailed Student’s t-test and *P < 0.05). (**C-D**) The vessels underwent immunofluorescence staining for indicated TAL1 and vWF, n = 5. L, lumen. Representative images are shown, Scale bar = 5 μm. (**E–F**) ApoE^–/–^ mice were injected with adeno-associated virus (AAV) encoding TAL1 or negative control AAV via the tail vein and fed a Western diet for four weeks.[41] Western blotting analysis of endothelial TAL1 protein levels in mouse vessels (n = 5 for each group, unpaired, two-tailed Student’s t-test and *P < 0.05). (**G-H**) Representative en face green fluorescent proteins and immunofluorescence staining of the expression of the TAL1 in ECs of the artery of mice, scale bar = 20 μm. (**I**) Representative en face preparations of whole aortas demonstrating atherosclerosis, visualized by Oil Red O staining. (**J**) Quantification of lesion area in the whole aorta (n = 5 for each group, unpaired, two-tailed Student’s t-test and *P < 0.05). (**K–M**) Hematoxylin and eosin and Oil Red O staining of the aortic root, scale bar = 100 µm. (n = 5 for each group, unpaired, two-tailed Student’s t-test and *P < 0.05)

We next determined the expression of TAL1 in atheroprone regions of human carotid arteries. And TAL1 protein levels were significantly reduced in atherosclerotic plaques compared to normal vessels (Fig. 7A, B). Furthermore, consistent with these results, TAL1 expression was lower in the intima of human carotid plaques compared to normal vascular (Fig. 7C, D). We investigated whether TAL1 over-expression could decrease atherosclerosis in vivo. ApoE^–/–^ mice were injected with endothelial-specific overexpression adeno-associated virus(AAV) vectors encoding TAL1 via the tail vein. TAL1 expression was markedly increased in the endothelial cells of AAV-TAL1 mice two weeks after injection compared to that in the AAV-EGFP mice (Fig. 7E–H). Endothelial TAL1 overexpression significantly reduced atherosclerotic lesions in the aorta (Fig. 7I, J). Moreover, both Oil Red O staining and H&E staining showed a significant reduction in plaque area at the aortic roots (Fig. 7K–M). VCAM-1 expression levels in the aortic root region of AAV-TAL1 mice were significantly reduced compared to control groups(Supplemental Fig. 10A-B). Taken together, these findings demonstrated the protective role of TAL1 in endothelial cells against atherosclerosis.

In conclusion, remdesivir limits endothelial cell activation by reducing LOX-1 expression and ROS production, and then promotes the binding of the core domain of TAL1 (186-331aa) to the C-terminal of TRAF6 in endothelial cells, followed by inhibiting the K63- linked polyubiquitination of TRAF6 and the activation of NF-κB. When TAL1 was silenced in endothelial cells, the K63 polyubiquitination of TRAF6 was enhanced and endothelial cells were spontaneously activated. Finally, over-expression TAL1 specifically in endothelial cells decreased inflammatory response and progression of atherosclerosis in ApoE-/- mice fed with western diet (Supplemental Fig. 12).

## Discussion

Remdesivir, an adenosine nucleotide analog prodrug, treats COVID-19 by inhibiting its progression and reducing patient recovery time [32]. Its effectiveness in alleviating acute kidney injury and non-alcoholic fatty liver disease highlights its potential for treating non-infectious inflammatory conditions [19]. However, the effect of remdesivir on atherosclerosis is still unclear. Our study illustrated that remdesivir decelerated atherosclerosis progression. This was achieved by diminished production of ROS triggered by ox-LDL in HUVECs, reinstating TAL1 protein levels, and facilitating the direct interaction between TAL1 and TRAF6. This decreased K63 ubiquitination of TRAF6, and its total ubiquitination reduced endothelial cell activation.

LOX-1 is a transmembrane glycoprotein serving as the primary receptor for ox-LDL in endothelial cells. It predominantly relies on its hydrophobic tunnel and hydrogen bonds formed by specific backbone and side-chain residues [27]. We observed that ox-LDL-induced LOX-1 activation enhanced Rac1-dependent NADPH oxidase activity, increasing ROS production [28]. However, remdesivir effectively decreased LOX-1 expression and ROS generation. Molecular docking studies revealed that remdesivir interacted with LOX-1 via hydrophobic bonds, suggesting that remdesivir may attenuate ox-LDL-LOX-1 interactions through an indirect competitive mechanism, potentially by modulating receptor accessibility or ligand-binding kinetics rather than direct steric competition.

In this study, ApoE (−/−) mouse model is widely used in atherosclerosis research due to its ability to spontaneously develop hyperlipidemia and atherosclerotic lesions resembling human pathology. While the differences in lipoprotein profiles (elevated VLDL-C and chylomicron remnants in mice vs. LDL-C/Lp(a)-driven disease in humans) pose translational limitations, the model’s lipid profile (enriched in oxidized VLDL remnants) still activates LOX-1, making it relevant for studying receptor-mediated inflammation, endothelial dysfunction, and foam cell formation.The precise mechanism by which remdesivir modulates ox-LDL-activated LOX-1 remains to be elucidated in upcoming research.

TAL1, also known as SCL, is a critical transcription factor in the hematopoietic system. It plays a pivotal role in blood development, survival, and dormancy of hematopoietic stem cells and the maturation of selected blood lineages [21]. SARS-CoV-2 upregulates TAL1 expression in mononuclear cells, an effect notably mitigated by remdesivir, [24] suggesting that remdesivir may exert its effects by regulating TAL1 expression. In vivo findings exposed that endothelial-specific overexpression of TAL1 in ApoE^–/–^ mice significantly impeded the aortic plaque formation, indicating the role of TAL1 in endothelial cells against atherosclerosis.

The unconventional E3 ubiquitin ligase TRAF6 is pivotal for regulating immune system function and homeostasis [9]. Its interaction with CD40 is critical in the context of atherosclerosis and restenosis [33]. Previous studies have indicated that the polyubiquitination of TRAF6 is closely related to TRAF6 activation. This autoubiquitination, particularly K63 ubiquitination, activates TAK1, subsequently activating IKK and NF-κB pathway [29]. Consistent with previous research, ox-LDL stimulated ROS production in endothelial cells, triggering the K63 ubiquitination of TRAF6 and TAK1 activation. Remdesivir effectively reduced ROS levels in endothelial cells, diminishing TRAF6 activation. Silencing TRAF6 attenuated the influences of ox-LDL on endothelial activation. This evidence emphasizes the TRAF6 as a key driver of endothelial cell activation in atherosclerosis. However, the specific binding and dissociation mechanism between TAL1 and TRAF6 has not been revealed yet. Whether TAL1 could affect the TRAF6 phosphorylation site and regulate the ubiquitination modification of TRAF6 requires further investigation.

So, this is the first study to provide evidence that remdesivir is an essential regulator of endothelial activation and atherosclerosis. Our research provides a novel pharmacological approach to prevent atherosclerosis.

## Materials and methods

### Key reagents

Human ox-LDL (IO1300, Solarbio); remdesivir (GS-5734) (S8932, Selleck); acetylcysteine (NAC) (S1623, Selleck); Lipofectamine™ 3000 (L3000008, Thermo); Reactive Oxygen Species Assay Kit (CA1410, Solarbio); BCECF-AM (pH fluorescent probe, 5 mM) (S1006, Biotium); Total RNA Extraction Kit I (R6834-01, OMEGA); PrimeScript RT reagent Kit with gDNA Eraser (RR047A, Takara); TB Green Premix Ex Taq II (RR820A, Takara); Anti-Myc Magnetic Beads (B26301, Selleck); Anti-Flag Magnetic Beads (B26101, Selleck);VCAM1 (E-10) (sc-13160, Santa Cruz); ICAM-1 (G-5) (sc-8439, Santa Cruz); LOX-1 (11837-1-AP, Proteintech); CD36 (18836-1-AP, Proteintech); SR-A1(sc-166184, Santa Cruz); Caspase-3(YM8058, Immunoway); cleaved-Caspase-3(YM8294, Immunoway); NF-κB p65 (D14E12) (8242, CST); Phospho-NF-κB p65 (Ser536) (93H1) (8242, CST); TRAF6 (D21G3) (8028S, CST), TRAF6 (D-10) (sc-8409, Santa Cruz); vWF(27186-1-AP, Proteintech); TAL1 (ab155195, Abcam); K63-linkage Specific Polyubiquitin (D7A11) (5621S, CST); Ubiquitin (E6K4Y) (20326S); HA-Tag (C29F4) (3724S, CST); DYKDDDDK Tag (D6W5B) (14793S, CST); Myc-Tag (9B11) (2276S, CST); Dylight594, goat anti-mouse IgG (H + L) (E032410-01, EarthOx); Dylight594, goat anti-rabbit IgG (H + L) (E032420-01, EarthOx); Dylight488, goat anti-rabbit IgG (H + L) (E032220-01, EarthOx); goat anti-rabbit IgG (H + L) HRP (S0001, Affinity); and goat anti-mouse IgG (H + L) HRP (S0002, Affinity);

### Animal experiments

Eight-week-old male ApoE^–/–^ mice were sourced from Beijing Hua Fu Kang Bioscience Co., Inc. All mice were acclimated for one week in a controlled temperature and humidity environment, maintained on a 12-h light–dark cycle, fed a Western diet with free access to food and water. Remdesivir (purity > 99.5%) was obtained from GLPBIO (USA), dissolved and stored in DMSO. Mice were fed with a Western diet (Research Diets, Cat No. D12109C) containing 40 kcal% fat, 1.25% cholesterol, and 0.5% cholic acid and injected i.p. with remdesivir (15 mg/kg) or vehicle (5%DMSO + 95%Corn oil) every 2 days for 4 weeks [34]. The AAV vector titers (viral genomes per mL) were determined by quantitative real-time PCR. For the AAV in vivo experiments, tail vein injections were administered with 2.5 × 10^12^ viral genomes (vgs) in a final volume of 100 μL.

### Adeno-associated virus (AAV) vector construction

Mouse Tal1 (FT2A-NM_011527-3FLAG) was obtained from the cDNA library of Genechem (Shanghai, China) with the following primers: Tal1 forward:5’‐ ACGAGCTGTACAAGGCTAGCATGCGGCGGAAGCGGGGCAGCGGCGAGGGCAGAGGAAGTCTTCTAAC‐3’ and reverse: 5’‐ GCCTCAGCTATTTAAAGCTTTCATTTGTCGTCATCATCCTTATAGTCCTTATCATCGTCGTCTTTGTAATCCTTGTC‐3’. The AAV vector plasmid GV652 (pAAV-ICAM2p-EGFP-MCS-SV40 PolyA, Serotype: ENT) (purchased from Shanghai Genechem Co., Ltd.), the vector and Tal1 gene sequence were digested by NheI and HindIII restriction enzymes,and complete cloning through In-fusion recombination method. Recombinant vector was detected by DNA sequencing.The viral vector were transfacted into 293 T cells using Lipofectamine 2000 (Invitrogen; Thermo Fisher Scientific, Inc.) together with plasmids pHelper and pRepCap.Adeno-Associated Virus were harvested 72 h post-transfection, AAV were purified through iodixanol gradient ultracentrifuge and subsequent concentration.Purified AAV viruses were tittered using a quantitative PCR-based method.All AAV used in this study was prepared in 0.001% Pluronic F-68 solution (Poloxamer 188 Solution,PFL01-100ML,Caisson Laboratories,Smithfield,UT,USA).

### Atherosclerotic lesion analysis

Histological analyses were performed on fresh-frozen and optimal cutting temperature compound-embedded aortic sections. Slides were fixed in 4% polyunsaturated fatty acid for 15 min, followed by H&E and Oil Red O staining. Images were captured by microscopy. For atherosclerotic lesion quantification in the aortic root, we collected 5–8 serial cross-section (7 μm thick) per mouse at 40–50 μm intervals, ensuring representative aortic sinus sampling. Sections were alternately used for H&E and Oil Red O staining. All sectional measurements per animal were averaged, with maximal lesion area included for statistically representative analysis.

### Cell culture and transfection

HUVECs were cultured as described [35]. HUVECs and HAECs were grown in the Endothelial Cell Medium (ECM, ScienCell Research Laboratories, Carlsbad, CA, USA) supplemented with 10% fetal bovine serum (FBS), 1% (v/v) penicillin/streptomycin, and 1% endothelial cell growth factors at 37 °C with 5% CO2 and 95% air. Cell passages 3 to 6 were used in all experiments. They were treated with 100 µg/mL ox-LDL with or without 10 µmol remdesivir for 24 h. HEK293T and THP-1 cells were cultured in RPMI 1640-medium containing 10% fetal bovine serum and 1% penicillin/streptomycin at 37 °C in a humidified atmosphere of 95% air/5% CO_2_. The medium was changed every two days.

HUVECs were transfected with 100 nM of control small interfering RNA (siRNA), LOX-1 siRNA, TRAF6 siRNA, and TAL1 siRNA (All four siRNA mentioned above were constructed by Keyybio, Shandong, China) for 24 h. FLAG-TAL1(Flag-tagged TAL1 were constructed by Keyybio, Shandong, China), MYC-TRAF6, HA-Ub, HA-K48-Ub and HA-K63-Ub(All four plasmids mentioned above were donated by Dr. Ding Ai (Tianjin Medical University, China)) were introduced into HEK293T cells. Transfection was performed using Lipofectamine 3000 or Lipofectamine RNAi Max, following the manufacturer’s instructions.

### Isolation and primary culture of mouse aortic endothelial cells

Mouse aortic endothelial cells were isolated as described [36–38]. Alternatively, Mice were anesthetized and euthanized, and the aorta was isolated and immersed in pre-chilled PBS. After removal of surrounding adipose and connective tissues, the cleaned aorta was transferred to a culture dish. A 1 mL syringe fitted with a 25G needle was inserted into one end of the aorta, and the lumen was gently flushed with ice-cold PBS to remove residual blood. The aorta was longitudinally incised using microdissection scissors to fully expose the intimal surface, followed by enzymatic digestion with 0.25% trypsin for 1 min at room temperature. Endothelial cells were gently scraped from the intimal surface using microforceps or a fixed circular needle, and the harvested material was collected into centrifuge tubes containing PBS. After centrifugation at 300–500 × g for 5 min, the supernatant was discarded, and the pellet was resuspended in complete cell culture medium. Cultures were maintained at 37 °C under 5% CO₂, and the medium was replaced after 24 h to remove non-adherent cells. Subculture was initiated when cells reached 80–90% confluence. All procedures were performed under strict sterile conditions, with precise control of enzymatic digestion time and centrifugation parameters to optimize cell viability and adherence.

### Quantitative real-time polymerase chain reaction (PCR)

Total mRNA was extracted using an Omega Bio-Tek kit. Furthermore, cDNA was synthesized using the PrimeScript RT reagent Kit with a gDNA Eraser (RR047A, Takara). Quantitative PCR (qPCR) was performed using TB Green Premix Ex Taq II (RR820A, Takara). All data were normalized to GAPDH expression levels.

### Immunofluorescence staining

Tissues or cell slides were fixed with 4% paraformaldehyde for 15 min, permeabilized with 0.05% Triton X-100 at room temperature for 15 min, and blocked with 5% bovine serum albumin for 30 min. Then incubated in 5% bovine serum albumin and a specific antibody incubation buffer at 4 °C for 36 h. After washing thrice with phosphate-buffered saline (PBS), the slides were incubated with Alexa Fluor 488- or Alexa Fluor 594-conjugated secondary antibody (EarthOx, USA) for 1 h at room temperature. The slides were then mounted with a 4′,6-diamidino-2-phenylindole (DAPI)-containing mounting medium, and representative images were taken from each group.

### Western blotting

Cells and tissues were lysed using cold radioimmunoprecipitation assay lysis buffer (Solarbio) containing a complete protease inhibitor (Beyotime, China). Total protein concentration was quantified using the bicinchoninic acid protein assay kit (Beyotime, China). Proteins were separated by 10% sodium dodecyl sulfate–polyacrylamide gel electrophoresis (SDS-PAGE) and transferred to a nitrocellulose membrane (Bio-Rad, USA). Target proteins were detected using specific primary antibodies followed by incubation with HRP-conjugated secondary antibodies. Protein bands were visualized using an enhanced chemiluminescence reagent in a Tanon Imaging System (Shanghai, China). By measuring integrated density, protein levels were quantified using NIH Image J software (https://imagej.nih.gov/ij/).

### Immunoprecipitation

Cell lysates were prepared as previously described [39]. For immunoprecipitation, 500 µg of protein was incubated with specific antibodies at 4 °C for 12 h. It was followed by adding 50 µL of 50% protein A/G agarose beads and then incubated at room temperature for 2 h. After washing five times with lysis buffer, the supernatant was discarded, and the precipitated proteins were resuspended in 2 × SDS-PAGE loading buffer, boiled for 5 min, and eluted from the beads. The bound proteins were detected by Western blotting.

### Cell adhesion assay

After treating HUVECs with ox-LDL and remdesivir for 24 h, THP-1 cells were labeled with BCECF-AM (Solarbio) and seeded onto HUVECs. After incubation at 37 °C for 1 h, the non-adherent cells were washed thrice with PBS. The number of adherent cells in five random fields was counted using a fluorescence microscope.

### Measurement of oxidative stress

Treated HUVECs were washed once with PBS and incubated with 10 µM Dihydroethidium(DHE) dye in the dark at 37 °C for 1 h. After washing thrice with PBS, the HUVECs were collected and analyzed using a flow cytometer (USA).

### Molecular docking

AutoDock4.2.6 was used for molecular docking of remdesivir with human OLR1 to obtain possible conformations and orientations of the ligand at the binding sites. The crystal structure of human OLR1 (PDB code: 6TL9) was used as a receptor. The grid parameter file of the binding pocket was created with the grid center 3.775, –47.516, and 45.252 (x, y, and z in Å) and dimensions 36 × 30 × 30 Å referring to the location of the binding ligand in 6TL9. The conformation with the lowest binding free energy was identified as the most probable. The interaction between the protein–ligand complex was mapped using PyMol.

Protein–protein docking in ClusPro3 was used for the molecular docking simulation of TAL1 with TRAF6 to obtain complex models. For the protein docking of TAL1 and TRAF6, the predicted model of TRAF6 was set as the receptor and TAL1 as the ligand. The ligand was rotated 70,000 times. The ligand was translated along the x-, y-, and z-axes relative to the receptor on a grid for each rotation. One translation with the best score was selected for each rotation. Of the 70,000 rotations, 1000 rotation/translation combinations with the lowest score were chosen. Then, greedy clustering of these 1000 ligand positions with a 9 Å C-alpha RMSD radius was performed to find the ligand positions with the most “neighbors” in 9 Å, such as cluster centers. The intermolecular contacts from the most probable poses were evaluated.

### Statistical analysis

According to the literature and previous studies, the sample sizes were designed with adequate power [40]. No sample outliers were excluded. Data are presented as the mean ± standard error of the mean, where n is the number of animals or cell cultures tested. The data were tested for normality before parametric statistics using the Shapiro–Wilk normality test (N < 10). Multiple tests were not conducted across all hypotheses tested. For normally distributed data, two groups were compared using an unpaired Student’s t-test. Comparisons among three or more groups were performed using one-way or two-way analysis of variance (ANOVA) followed by post-hoc Bonferroni correction. Non-normal data comparisons were performed using the Mann–Whitney U test or the Kruskal–Wallis test, followed by Dunn’s multiple comparison test. The exact test used for each dataset is described in Figure Legends. In all experiments, *P* < 0.05 was considered statistically significant. Each *P*-value is provided in the Figures. Statistical analysis was performed using Graph Pad Prism software (version 9.0; Graph Pad Software lnc).

## Supplementary Information

Additional file 1

Additional file 2

## Data Availability

Data collected in this study are available from the corresponding author upon reasonable request.

## Abbreviations

VCAM-1: Vascular cell adhesion molecule 1
ICAM-1: Intercellular adhesion molecule 1
TRAF6: TNF receptor-associated factor 6
TAL1: T-cell acute lymphoblastic leukemia 1
THP-I: Human monocytic leukemia 1
GAPDH: Glyceraldehyde 3-phosphate dehydrogenase
DMSO: Dimethyl sulfoxide
DAPI: 4′,6-Diamidino-2-phenylindole
H&E: Hematoxylin and eosin
HRP: Horseradish peroxidase
ox-LDL: Oxidized low-density lipoprotein
NF-κB: Nuclear factor kappa B
ROS: Reactive oxygen species
SDS-PAGE: Sodium dodecyl sulfate-Polyacrylamid gel electrophoresis
WB: Western blot
