# Supplementary material for: Remdesivir inhibits endothelial activation and atherosclerosis by coupling TAL1 to TRAF6
Source: J Transl Med. 2025 Jul 1;23:719. doi: 10.1186/s12967-025-06673-2 (PMC12220244; doi:10.1186/s12967-025-06673-2)
Supplement: Supplementary file 1 — Additional file 1 [file 12967_2025_6673_MOESM1_ESM.doc]

**Supplement to “Remdesivir inhibits endothelial activation and atherosclerosis by coupling TAL1 to TRAF6”**

Hanning Zhang^a,1^, Ruru Li^a,1^, Qianqian Huo^b^, Li Li^b^, Min Li^b^, Shunxin Hu^a^, Changjie Ren^b,c^, Zongyin Wu^b,c*^ Chenghu Zhang^b,c*^

a Shandong First Medical University, Jinan, China.

b Department of Cardiology, Jining First People's Hospital, Shandong First Medical University, Jining, China.

c Jining Key Laboratory of Metabolic Cardiovascular Diseases, Jining, China.

* Corresponding author.

Correspondence to: Chenghu Zhang, Department of Cardiology, Jining First People's Hospital, Shandong First Medical University, No. 6 Jiankang Road, Jining 272000, Shandong , China; Jining Key Laboratory of Metabolic Cardiovascular Diseases, Jining, China. Zongyin Wu, Department of Cardiology, Jining First People's Hospital, Shandong First Medical University, No. 6 Jiankang Road, Jining 272000, Shandong , China. Jining Key Laboratory of Metabolic Cardiovascular Diseases, Jining, China.

E-mail address: zchjnyy2020@163.com; wuzysp@163.com

1 These authors contributed equally to this work.

**
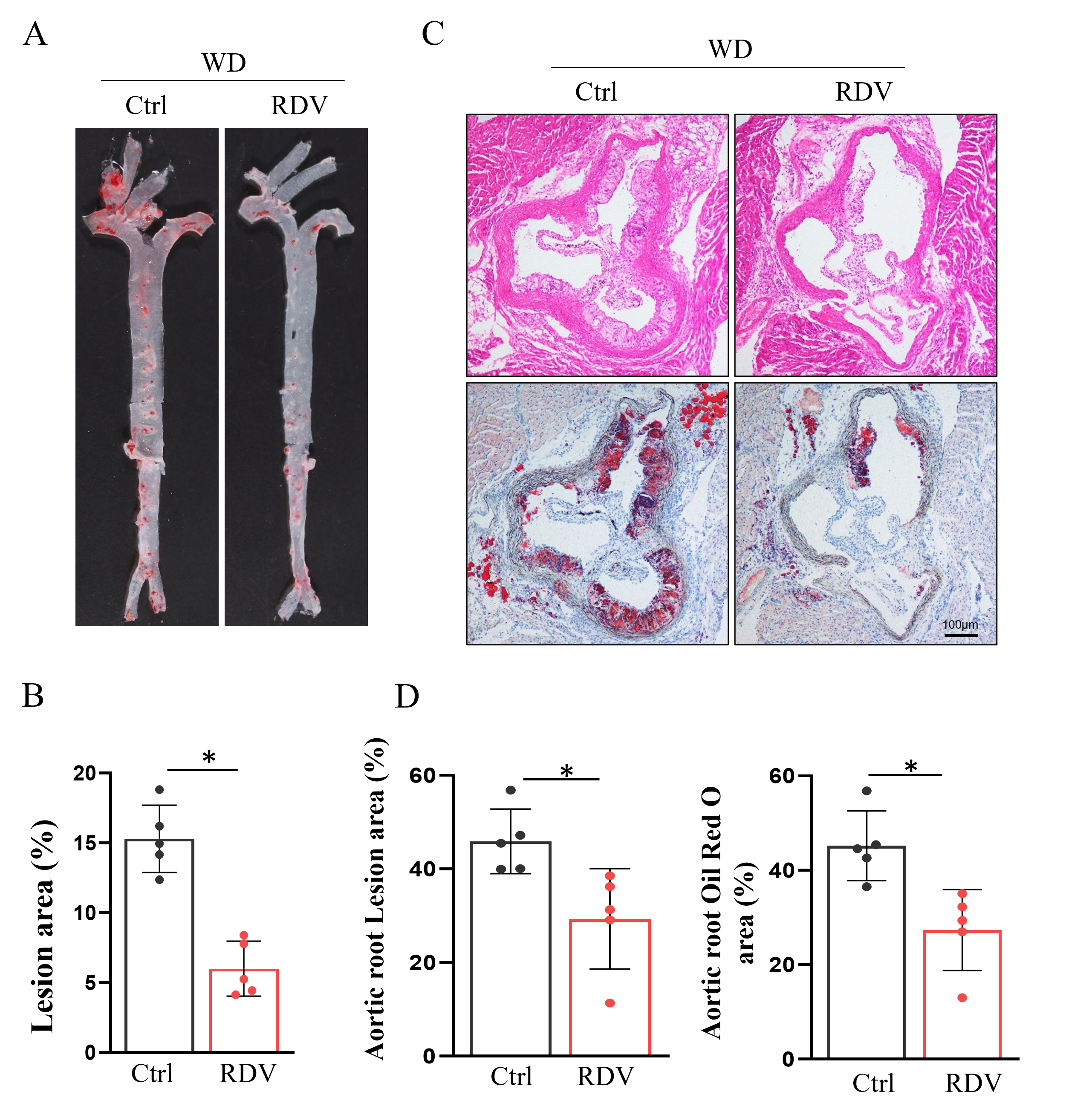
**

**
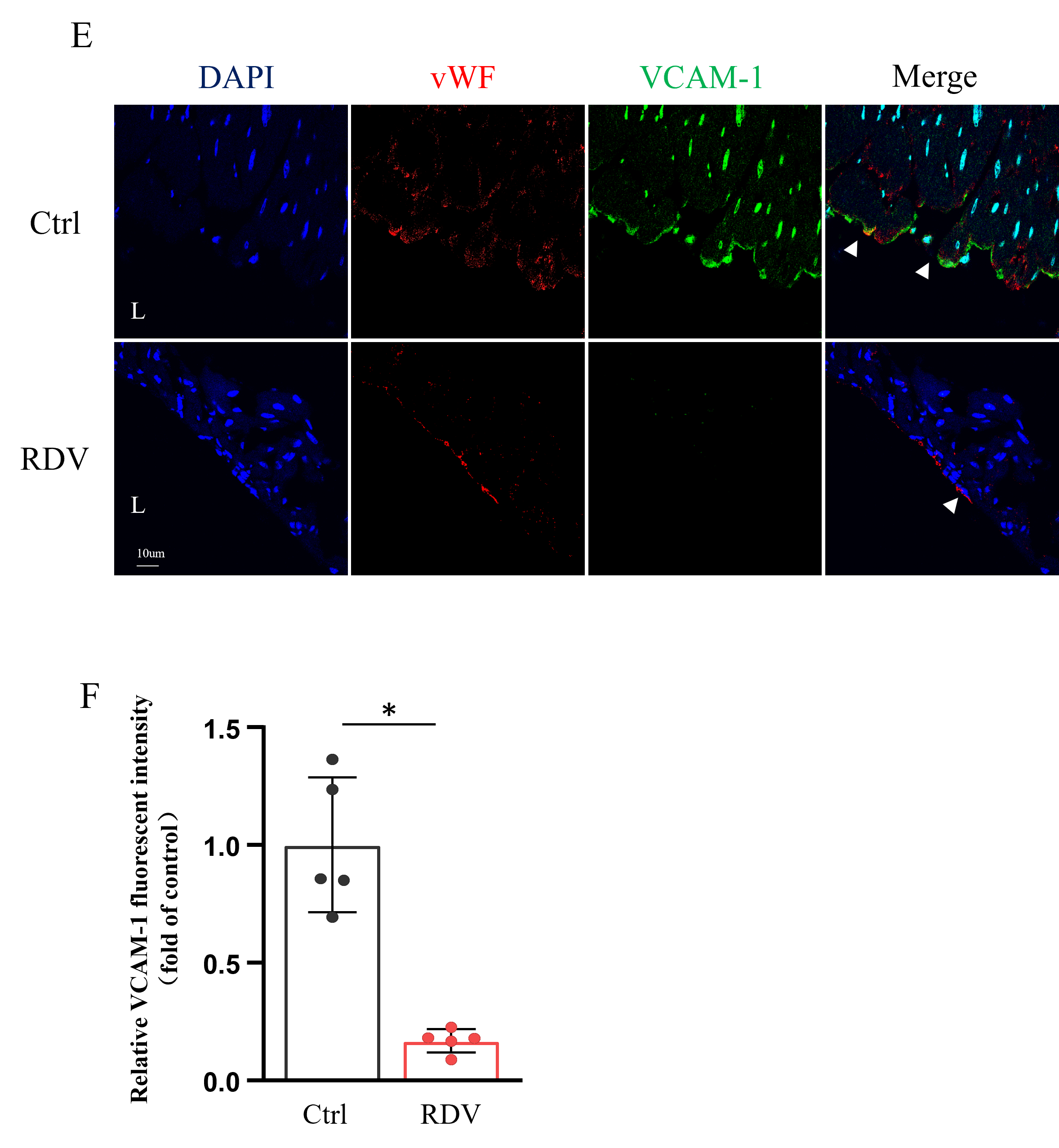
**

**Supplemental Figure 1. Remdesivir reduced diet-induced atherosclerosis in female ApoE−/− mice.** Female ApoE^–/–^ mice were treated with remdesivir (15 mg/kg) or DMSO every two days and fed a Western diet for four weeks. (A) Representative en face preparations of whole aortas representing atherosclerosis, visualized by Oil Red O staining. (B) Quantification of lesion area in the whole aorta (n = 5 for each group, unpaired, two-tailed Student’s t-test and **P* < 0.05). (C-D) Hematoxylin and eosin and Oil Red O staining of the aortic root, scale bar = 100 μm (n = 5 for each group, unpaired, two-tailed Student’s t-test and **P* < 0.05).(E-F) VCMA-1/von Willebrand factor (vWF) immunofluorescence staining of aortic roots and quantification of relative fluorescent intensity of VCAM-1 (normalized to WD+control group).L, lumen. Scale bar, 10μm. (n = 5 for each group, unpaired, two-tailed Student’s t-test and **P* < 0.05)

**
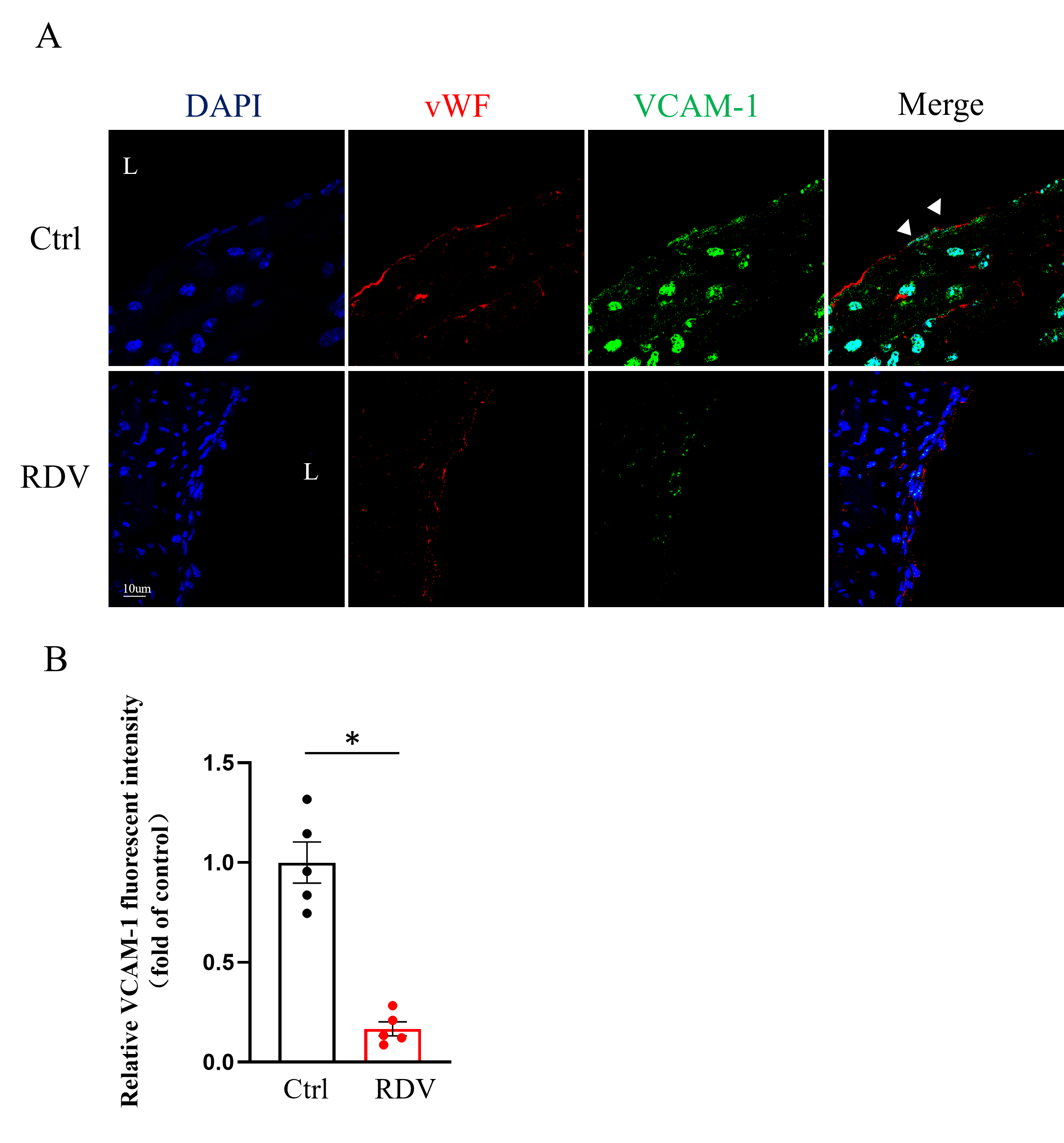
**

**Supplemental Figure 2. Remdesivir reduced diet-induced atherosclerosis in male ApoE−/− mice.**

(A-B) VCMA-1/von Willebrand factor (vWF) immunofluorescence staining of aortic roots and quantification of relative fluorescent intensity of VCAM-1 (normalized to WD+control group).L, lumen. Scale bar, 10μm. (n = 5 for each group, unpaired, two-tailed Student’s t-test and **P* < 0.05)

**
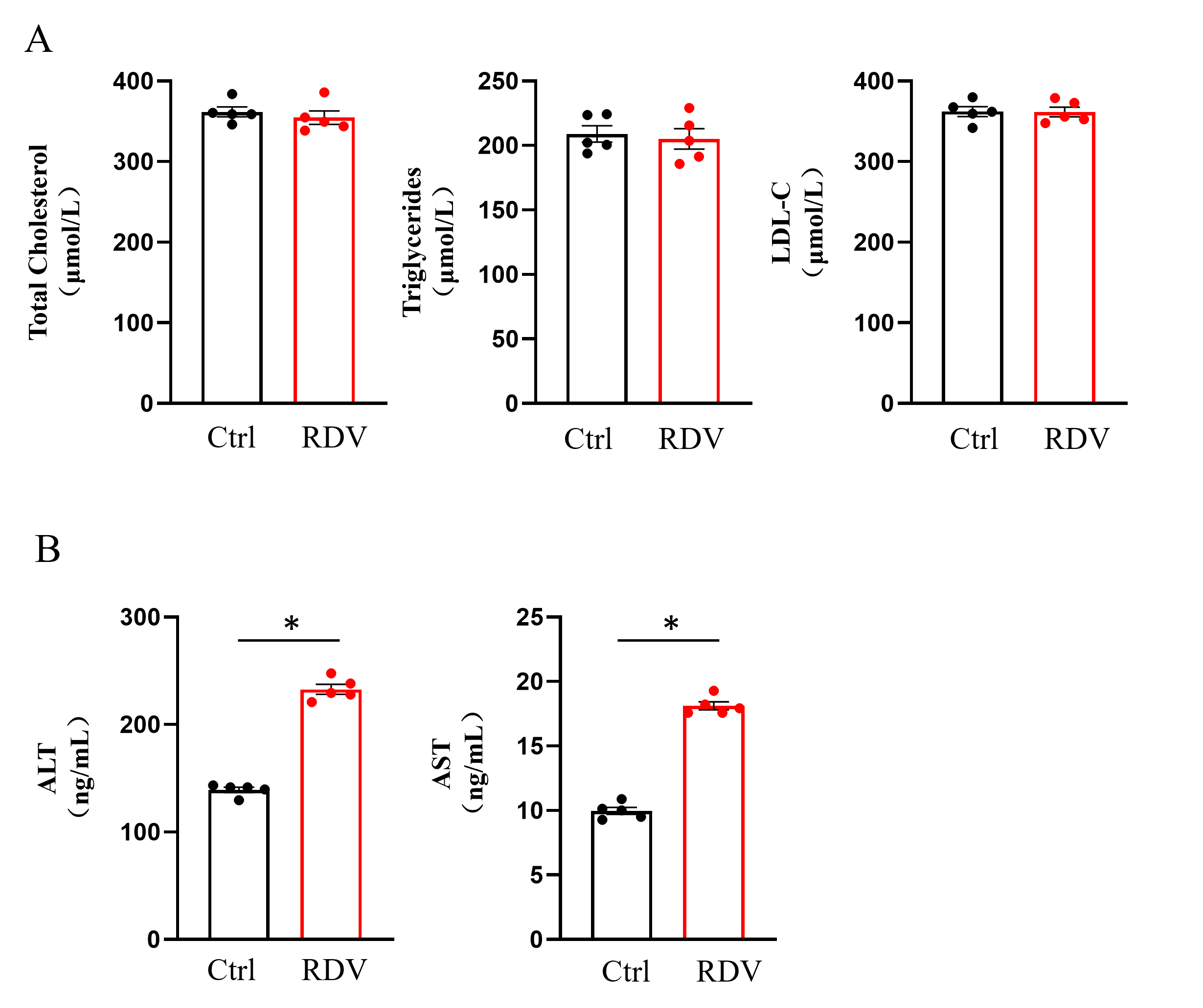
**

**Supplemental Figure 3. Remdesivir induces a mild elevation in transaminase levels without altering the lipid profile in mice.** Male ApoE^–/–^ mice were treated with remdesivir (15 mg/kg) or DMSO every two days and fed a Western diet for four weeks. Quantification of ALT、AST and plasma levels of triglycerides, total cholesterol, and low-density lipoprotein cholesterol (LDL-C) (at the beginning and the end of experiment). (n = 5 for each group, unpaired, two-tailed Student’s t-test and **P* < 0.05)

**
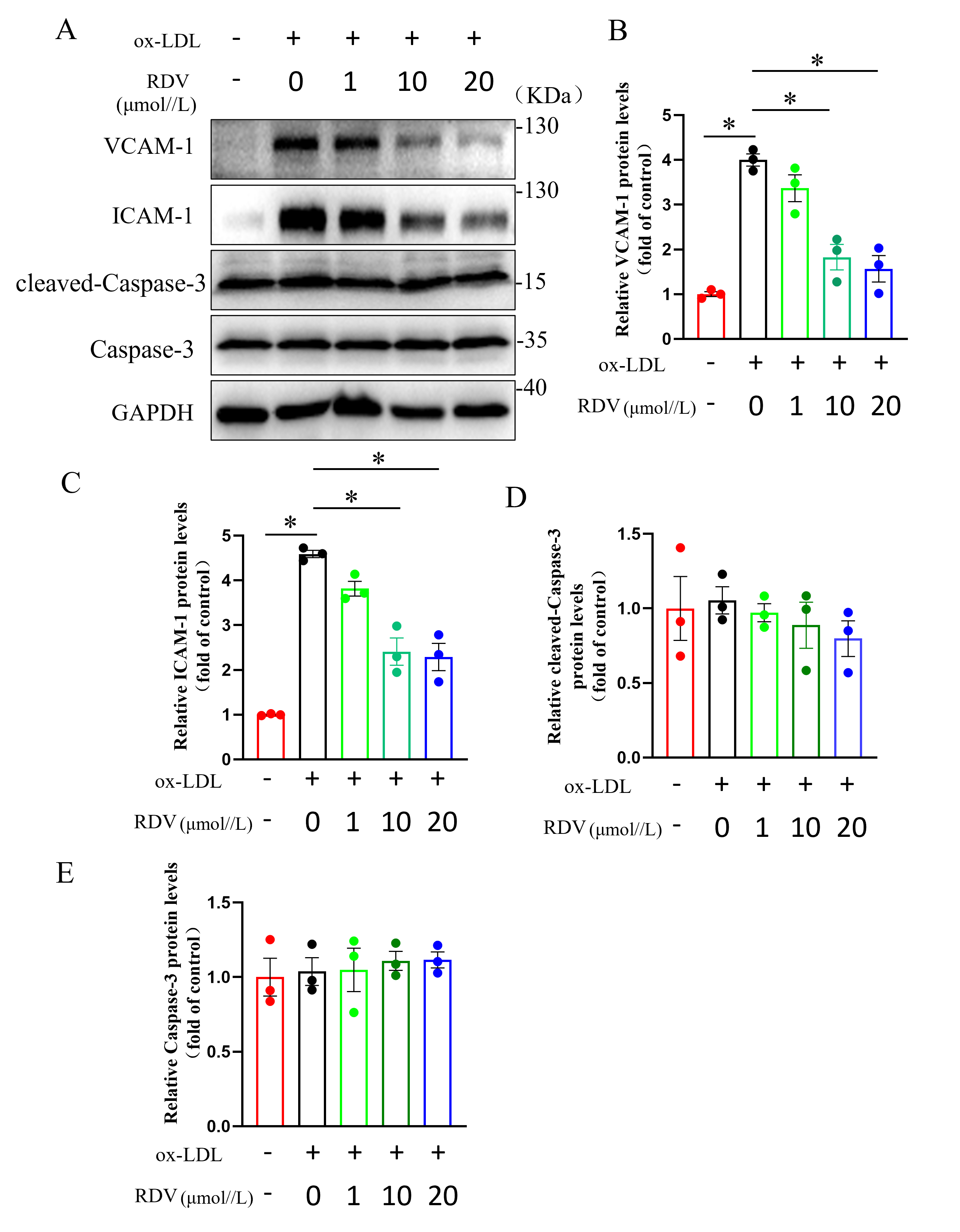
**

**Supplemental Figure 4. Remdesivir did not affect the cytotoxicity in HUVECs.** HUVECs were treated with ox-LDL (100 μg/mL) and remdesivir with indicated concentration for 24h, (A-C) Representative Western blots of VCAM-1 and ICAM-1; (D-E) Cell cytotoxicity was measured by caspase-3 and cleaved-casease3 assays. (n = 3 for each group, two-way ANOVA with Bonferroni multiple comparisons post-hoc tests and **P* < 0.05).

**
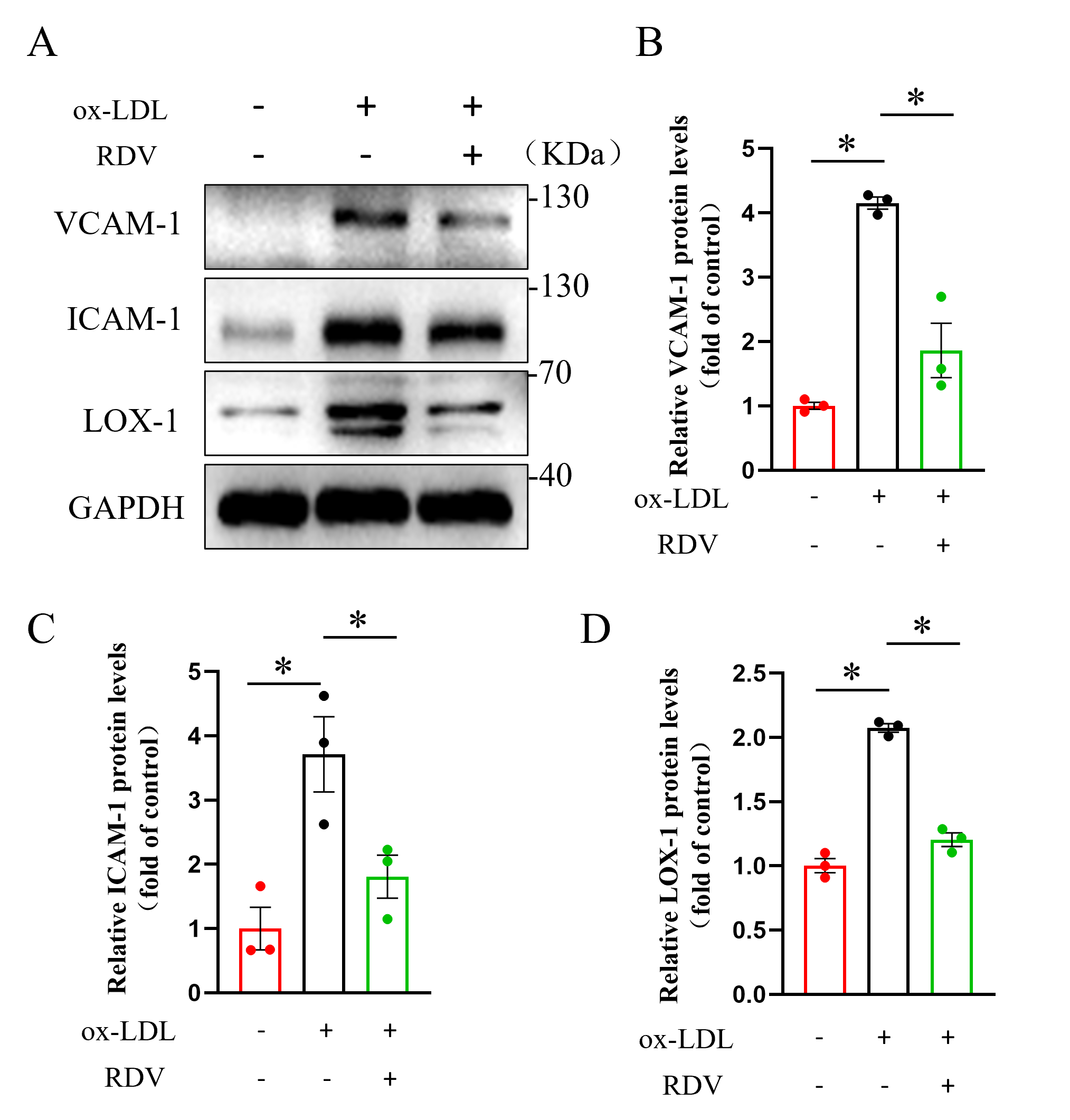
**

**Supplemental Figure 5. Remdesivir reduced endothelial cell activation in HAECs.** Treatment of HAECs with ox-LDL and remdesivir for 24 h, (A-D) Representative Western blots of VCAM-1、ICAM-1 and LOX-1 (n = 3 for each group, one-way ANOVA with Bonferroni multiple comparisons post-hoc tests and **P* < 0.05).

**
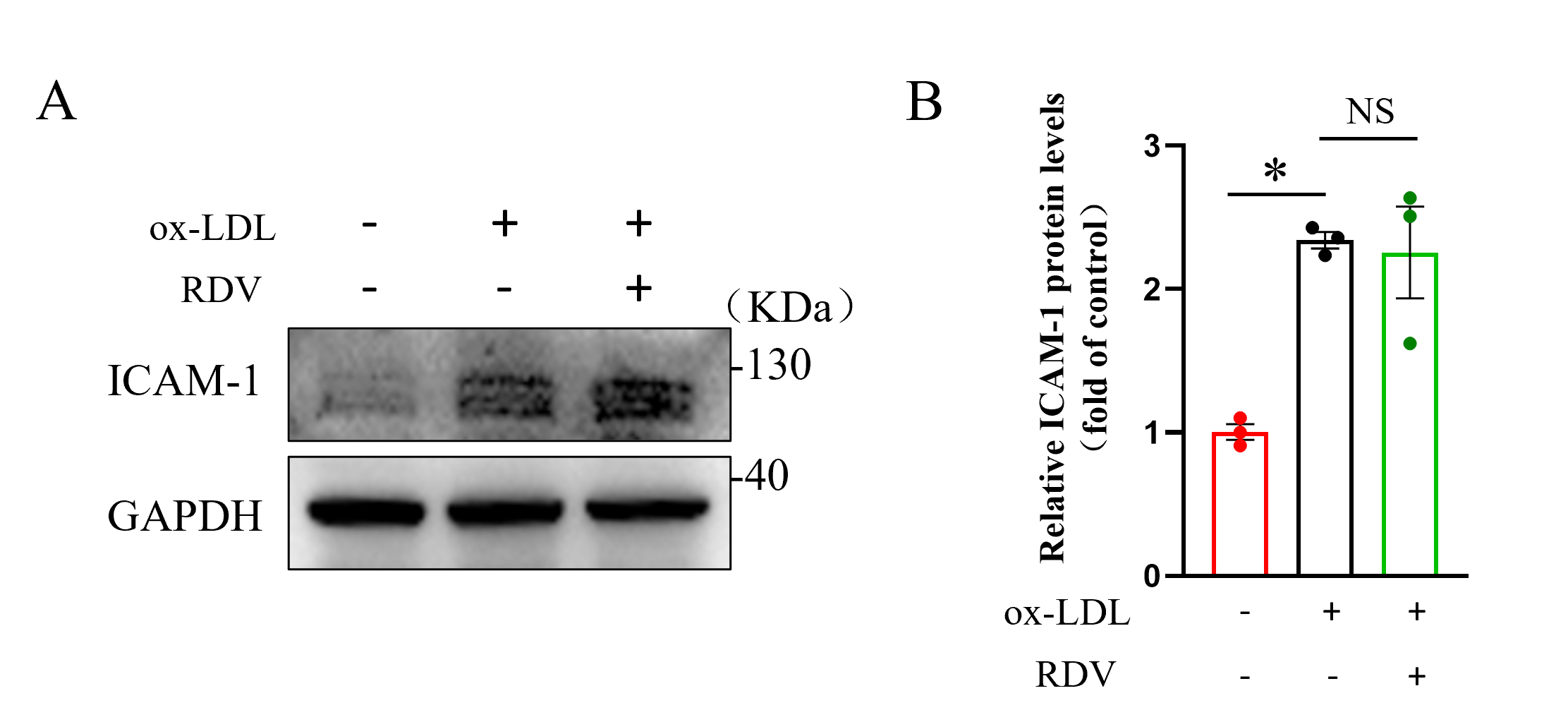
**

**Supplemental Figure 6. Remdesivir had no effect on macrophages.**Treatment of RAW264.7 macrophages with ox-LDL and remdesivir for 24 h, (A-B) Representative Western blots of ICAM-1 (n = 3 for each group, one-way ANOVA with Bonferroni multiple comparisons post-hoc tests and **P* < 0.05).

**
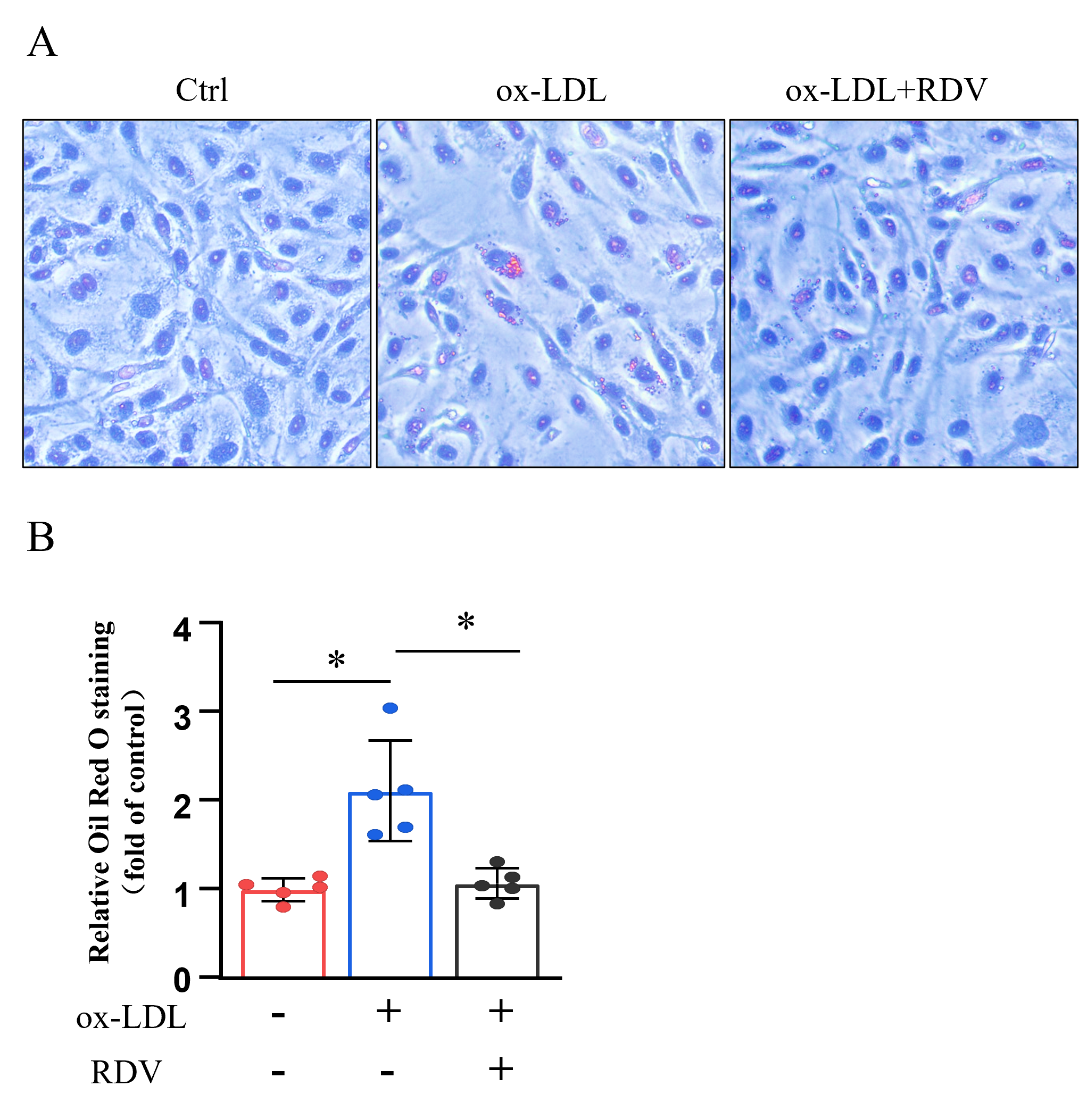
**

**Supplemental Figure 7. Lipid deposition of the HUVECs due to ox-LDL.** HUVECs were treated with either ox‑LDL and remdesivir for 24h, (A) Lipid accumulation was detected using Oil Red O staining. (B) Quantiffcation of neutral lipids was analysed using Fiji software. Magniffcation, x10. (n = 3 for each group, one-way ANOVA with Bonferroni multiple comparisons post-hoc tests and **P* < 0.05).

**
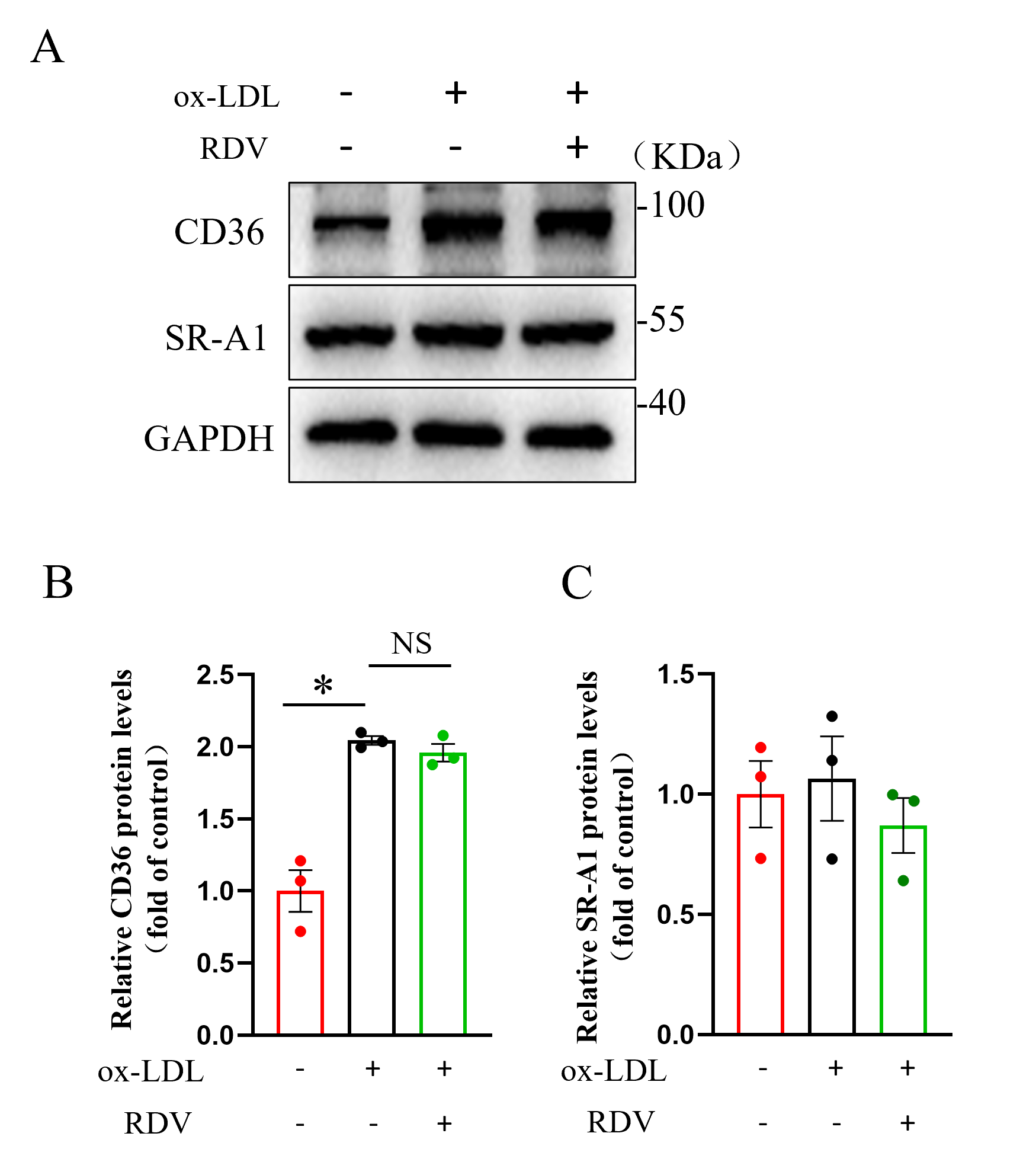
**

**Supplemental Figure 8. Remdesivir had no effect on CD36 and SR-A1.** Treatment of HUVECs with ox-LDL and remdesivir for 24 h, (A-C) Representative Western blots of CD36 and SR-A1 (n = 3 for each group, one-way ANOVA with Bonferroni multiple comparisons post-hoc tests and **P* < 0.05).

**
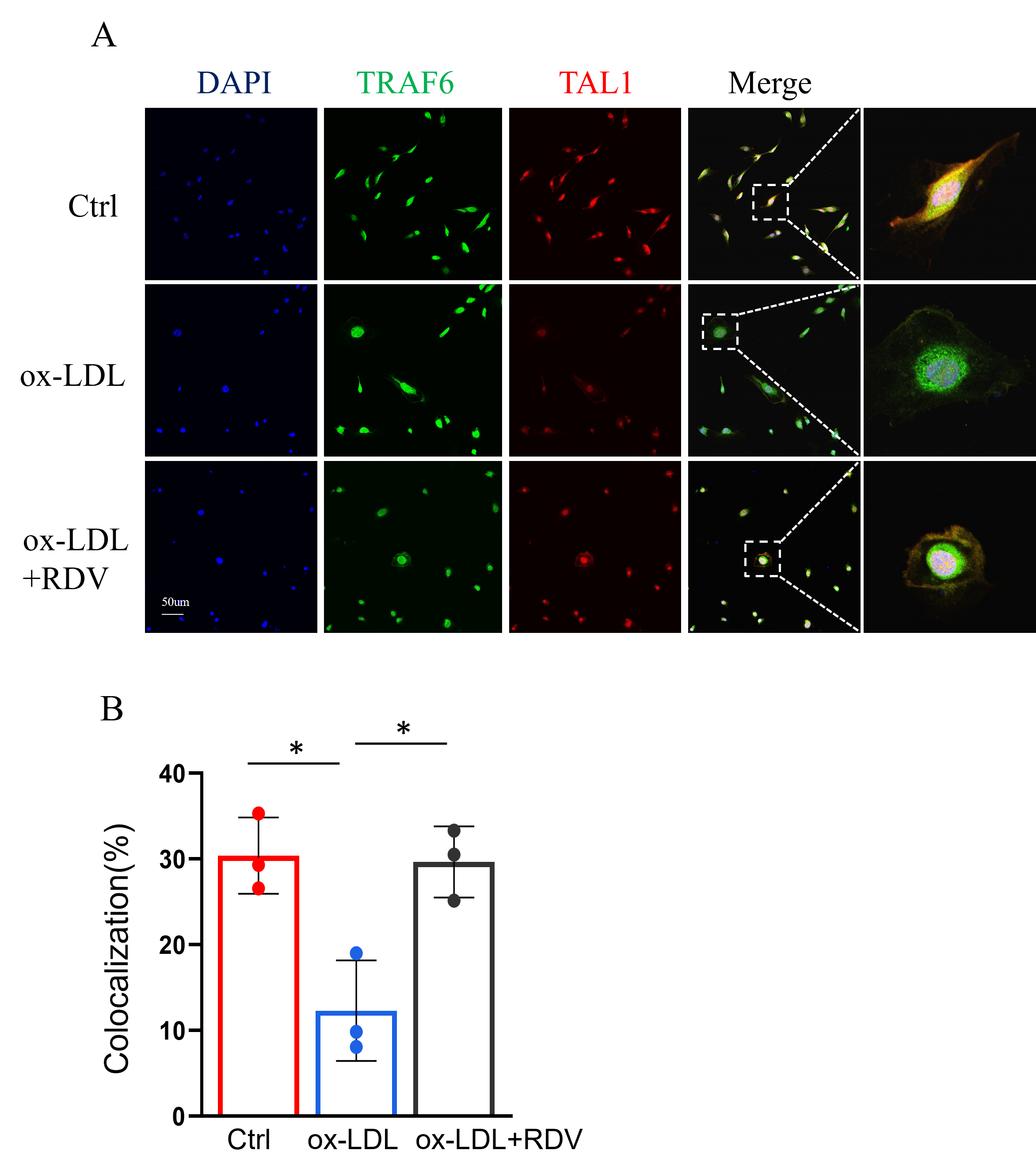
**

**Supplemental Figure 9. TAL1 Interaction with TRAF6.** (A-B) HUVECs were treated with ox-LDL and remdesivir for 24 h, immunofluorescence staining of TAL1 (red) and TRAF6 (green), and blue fluorescence indicating DAPI, scale bar =50μm. Quantification of co-localized relative fluorescence intensity in three random fields. (n = 3 for each group, one-way ANOVA with Bonferroni multiple comparison post-hoc test, *P < 0.05).

**
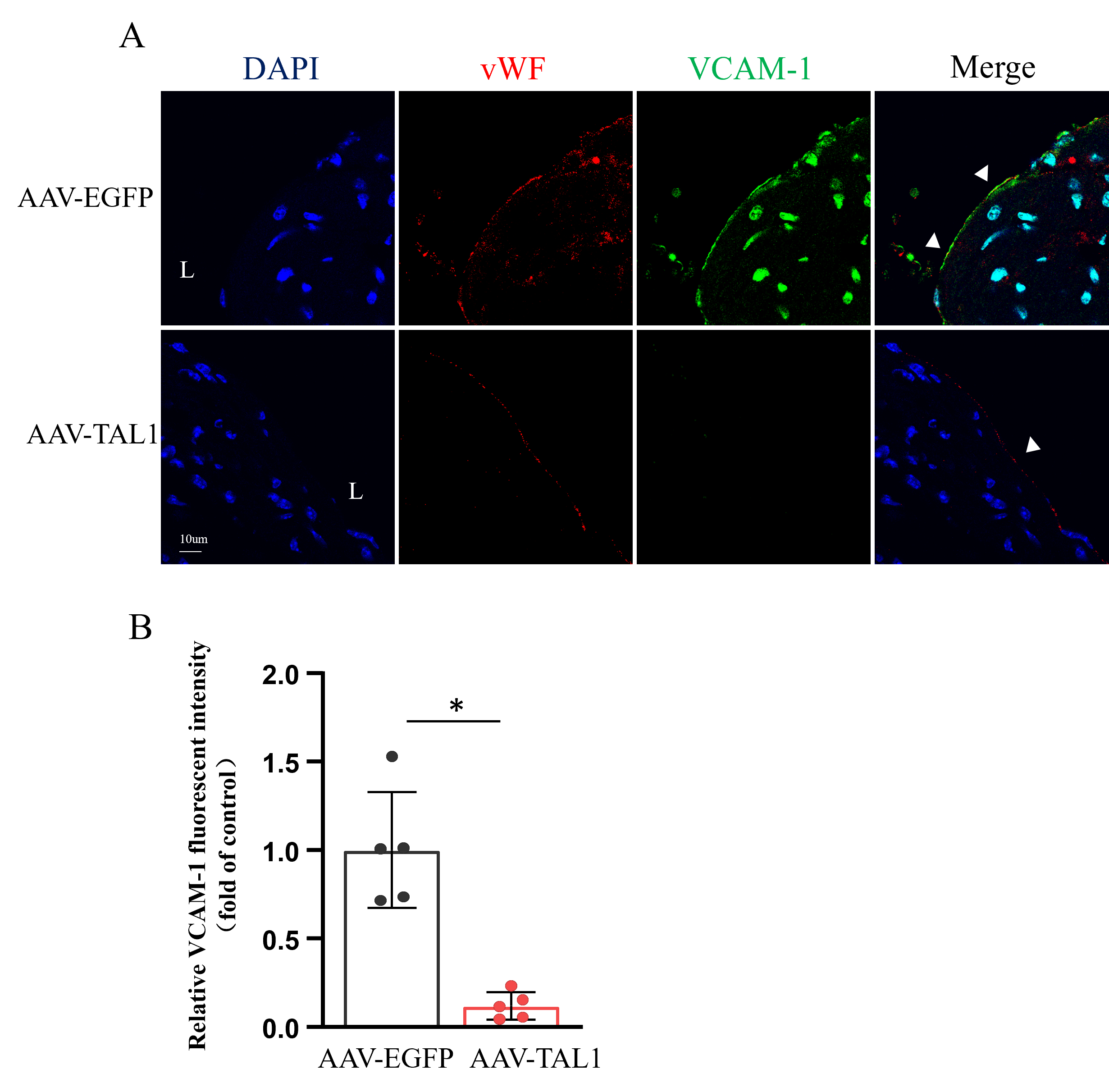
**

**Supplemental Figure 10. Endothelial-specific TAL1 overexpression decreased atherosclerosis.** (A-B) VCMA-1/von Willebrand factor (vWF) immunofluorescence staining of aortic roots and quantification of relative fluorescent intensity of VCAM-1 (normalized to WD+AAV-EGFP group). L, lumen. Scale bar, 10μm. (n = 5 for each group, unpaired, two-tailed Student’s t-test and **P* < 0.05)

**
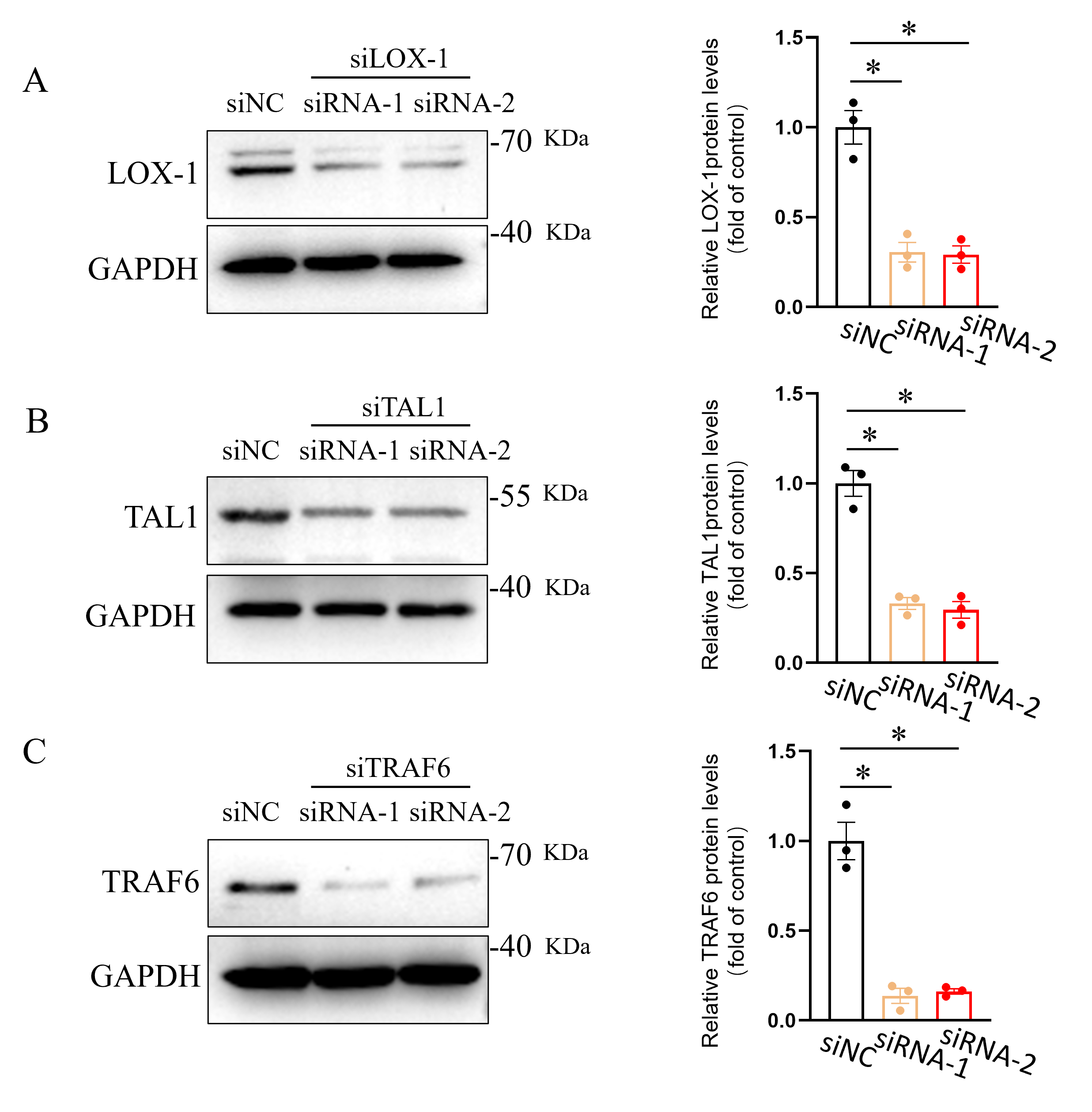
**

**Supplemental Figure 11. Dual siRNA Validation for Reliable Results.** (A-C) HUVECs were transfected with dual siRNA for 24 h. Quantification analysis of LOX-1, TAL-1, and TRAF6 by Western blotting (n = 3 for each group, one-way ANOVA with Bonferroni multiple comparisons post-hoc tests and *P < 0.05).


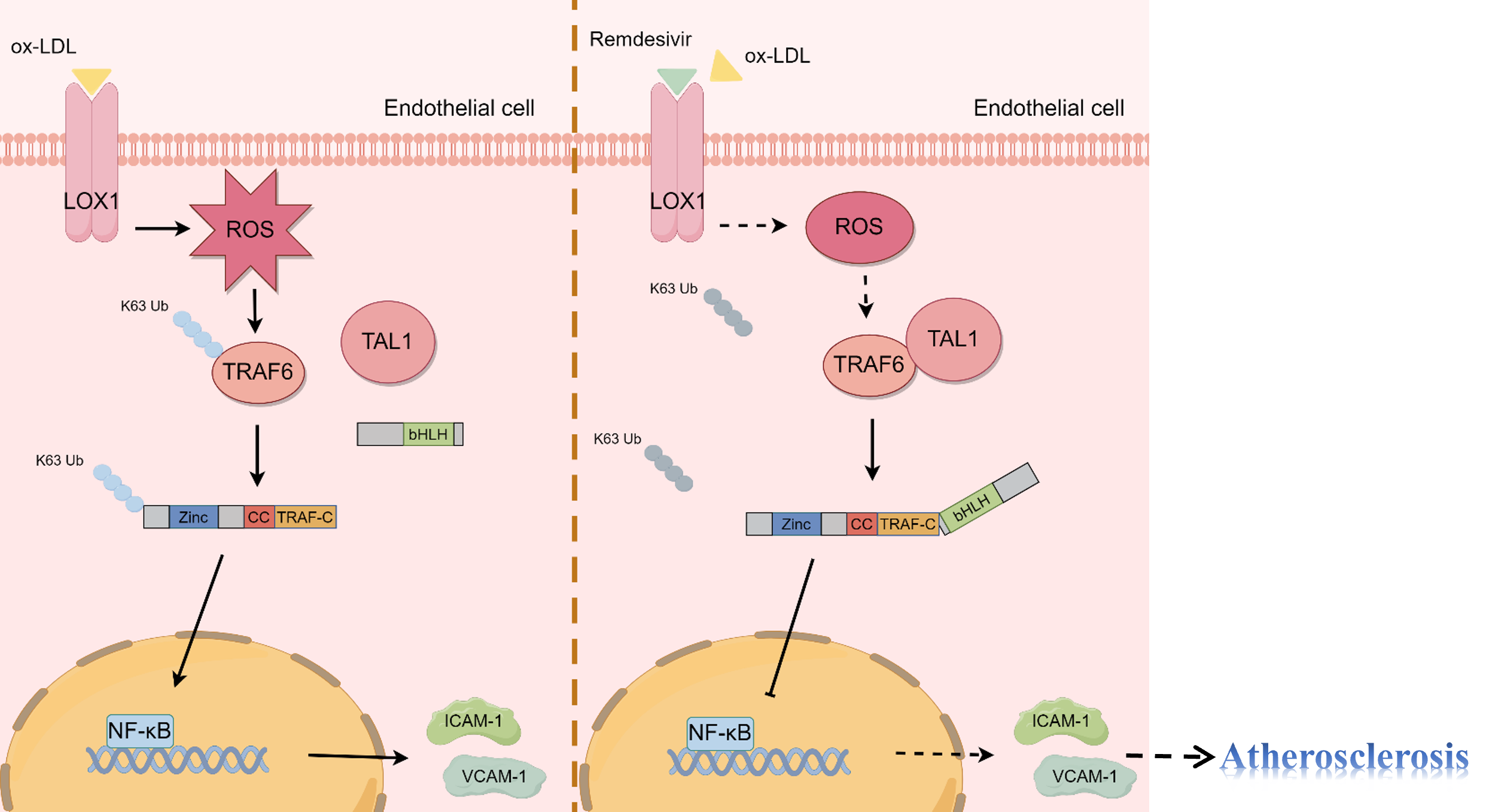


**Supplemental Figure 12. Schematic diagram depicting the key findings of this study**
