# Supplementary material for: Remdesivir inhibits endothelial activation and atherosclerosis by coupling TAL1 to TRAF6
Source: J Transl Med. 2025 Jul 1;23:719. doi: 10.1186/s12967-025-06673-2 (PMC12220244; doi:10.1186/s12967-025-06673-2)
Supplement: Supplementary file 2 — Additional file 2 [file 12967_2025_6673_MOESM2_ESM.docx]

**Supplemental Table 1. The contact list between TRAF6 and TAL1.**

| **Protein** | **Residue** | **Protein** | **Residue** | **Interaction type** |
| --- | --- | --- | --- | --- |
| TRAF6 | Asp161: OD1 | TAL1 | Gly114: CA | Hydrogen bond |
| TRAF6 | Asp161: OD1 | TAL1 | Asp115: N | Hydrogen bond |
| TRAF6 | Met272: SD | TAL1 | Gln252: N | Hydrogen bond |
| TRAF6 | Glu303: N | TAL1 | Gly250: O | Hydrogen bond |
| TRAF6 | Thr317: OG1 | TAL1 | Glu249: OE1 | Hydrogen bond |
| TRAF6 | Ala318: N | TAL1 | Glu249: OE1 | Hydrogen bond |
| TRAF6 | Thr322: OG1 | TAL1 | Tyr235: OH | Hydrogen bond |
| TRAF6 | Gln323: OE1 | TAL1 | Asn288: ND2 | Hydrogen bond |
| TRAF6 | Gly383: O | TAL1 | Thr305: CB | Hydrogen bond |
| TRAF6 | Lys384: NZ | TAL1 | Thr305: OG1 | Hydrogen bond |
| TRAF6 | Lys384: NZ | TAL1 | Glu306: O | Hydrogen bond |
| TRAF6 | Arg431: NH2 | TAL1 | Arg331: O | Hydrogen bond |
| TRAF6 | Glu498: OE1 | TAL1 | Arg331: C | Hydrogen bond |
| TRAF6 | Arg502: NH1, NH2 | TAL1 | Glu306: OE1 | Hydrogen bond, Salt bridge |
| TRAF6 | Arg516: NH1 | TAL1 | Leu322: O | Hydrogen bond |
| TRAF6 | Arg516: NH1 | TAL1 | Ala325: O | Hydrogen bond |
| TRAF6 | Arg516: CB | TAL1 | Asp326: OD2 | Hydrogen bond |
| TRAF6 | Ser517: OG | TAL1 | Arg331: NH2 | Hydrogen bond |

**Supplemental Table 2. Reagents and Antibodies**

**Reagents :**

| **Reagents** | **Product number** | **Source** |
| --- | --- | --- |
| Human ox-LDL | Cat#IO1300 | Solarbio |
| Remdesivir (GS-5734) | Cat#S8932 | Selleck |
| Acetylcysteine | Cat#S1623 | Selleck |
| Lipofectamine™ 3000 | Cat#L3000008 | Thermo Fisher |
| Lipofectamine RNAi MAX transfection reagent | Cat#13778150 | Thermo Fisher |
| Protein A/G PLUS-Agarose beads | Cat#20423 | ThermoFisher |
| RIPA buffer | Cat#R0010 | Solarbio |
| Reactive Oxygen Species Assay Kit | Cat#CA1410 | Solarbio |
| BCECF-AM (pH fluorescent probe, 5 mM) | Cat#S1006 | Biotium |
| Total RNA Extraction Kit I | Cat#R6834-01 | OMEGA |
| PrimeScript RT reagent Kit with gDNA Eraser | Cat#RR047A | Takara |
| TB Green Premix Ex Taq II | Cat#RR820A | Takara |
| Anti-Myc Magnetic Beads | Cat#B26301 | Selleck |
| Anti-Flag Magnetic Beads | Cat#B26101 | Selleck |
| Mouse TC ELISA Kit | Cat#MM-0632M2 | Jiangsu Meimian industrial Co., Ltd |
| Mouse TG ELISA Kit | Cat#MM-0631M2 | Jiangsu Meimian industrial Co., Ltd |
| Mouse LDL ELISA Kit | Cat#MM-44068M2 | Jiangsu Meimian industrial Co., Ltd |
| Mouse ALT ELISA Kit | Cat#MM-0260M2 | Jiangsu Meimian industrial Co., Ltd |
| Mouse AST ELISA Kit | Cat#MM-44115M2 | Jiangsu Meimian industrial Co., Ltd |

**Antibodies:**

| **Antibodies** | **Product number** | **Source** | **Concentration** |
| --- | --- | --- | --- |
| VCAM1 (E-10) | Cat#sc-13160 | Santa Cruz | 1:1000, Western blot; 1:100, Immunofluorescence |
| ICAM-1 (G-5) | Cat#sc-8439 | Santa Cruz | 1:1000, Western blot; 1:100, Immunofluorescence |
| LOX-1 | Cat#11837-1-AP | Proteintech | 1:1000, Western blot |
| CD36 | Cat#18836-1-AP | Proteintech | 1:1000, Western blot |
| SR-A1 | Cat#sc-166184 | Santa Cruz | 1:1000, Western blot |
| Caspase-3 | Cat#YM8058 | Immunoway | 1:1000, Western blot |
| cleaved-Caspase-3 | Cat#YM8294 | Immunoway | 1:1000, Western blot |
| NF-κB p65 | Cat#8242 | CST | 1:1000, Western blot |
| Phospho-NF-κB p65 | Cat#8242 | CST | 1:1000, Western blot |
| TRAF6 (D21G3) | Cat#8028S | CST | 1:1000, Western blot; Immunoprecipitation |
| TRAF6 (D-10) | Cat#sc-8409 | Santa Cruz | 1:1000, Western blot; Immunoprecipitation |
| vWF | Cat#27186-1-AP | Proteintech | 1:100, Immunofluorescence |
| TAL1 | Cat#ab155195 | Abcam | 1:1000, Western blot; Immunoprecipitation |
| TAL1 | Cat#sc-393287 | SantaCruz | 1:1000,Westernblot;1:100,Immunofluorescence;Immunoprecipitation |
| GAPDH | Cat#sc-47724 | SantaCruz | 1:1000, Western blot |
| K63-linkage Specific Polyubiquitin | Cat#5621S | CST | 1:1000, Western blot |
| Ubiquitin (E6K4Y) | Cat#20326S | CST | 1:1000, Western blot |
| HA-Tag (C29F4) | Cat#3724S | CST | 1:1000, Western blot |
| DYKDDDDK Tag (D6W5B) | Cat#14793S | CST | 1:1000, Western blot |
| Myc-Tag (9B11) | Cat#2276S | CST | 1:1000, Western blot |
| Dylight594, goat anti-mouse IgG (H+L) | Cat#E032410-01 | EarthOx | 1:100, Immunofluorescence |
| Dylight594, goat anti-rabbit IgG (H+L) | Cat#E032420-01 | EarthOx | 1:100, Immunofluorescence |
| Dylight488, goat anti-rabbit IgG (H+L) | Cat#E032220-01 | EarthOx | 1:100, Immunofluorescence |
| Dylight488, goat anti-mouse IgG (H+L) | Cat#E032210-01 | EarthOx | 1:100, Immunofluorescence |
| goat anti-rabbit IgG (H+L) HRP | Cat#S0001 | Affinity | 1:5000, Western blot |
| goat anti-mouse IgG (H+L) HRP | Cat#S0002 | Affinity | 1:5000, Western blot |

**Supplemental Table 3. List of gene-specific primer and siRNA sequences**

| **Gene(human)** | **Forward primer** | **Reverse primer** |
| --- | --- | --- |
| VCMA-1 | GGGAAGATGGTCGTGATCCTT | TCTGGGGTGGTCTCGATTTTA |
| ICAM-1 | ATGCCCAGACATCTGTGTCC | GGGGTCTCTATGCCCAACAA |
| LOX-1(OLR1) | TTGCCTGGGATTAGTAGTGACC | GCTTGCTCTTGTGTTAGGAGGT |
| GAPDH | GGAGCGAGATCCCTCCAAAAT | GGCTGTTGTCATACTTCTCATGG |
| **Gene(human)** | **Forward sequences** | **Reverse sequences** |
| TAL1(human）siRNA-109 | CGAGUGAAGAGGAGACCUUtt | AAGGUCUCCUCUUCACUCGtt |
| TAL1(human）siRNA-317 | AUAUCAACUUCUUGGCCAAtt | UUGGCCAAGAAGUUGAUAUtt |
| OLR1(human）siRNA-1595 | GGACAGAUUAAUAGAUUUATT | UAAAUCUAUUAAUCUGUCCTT |
| OLR1(human）siRNA-1771 | GACACUCAAUCUAGAACAATT | UUGUUCUAGAUUGAGUGUCTT |
| TRAF6(human）siRNA-1354 | GGAGAAACCUGUUGUGAUUTT | AAUCACAACAGGUUUCUCCTT |
| TRAF6(human）siRNA-409 | GGGAUAUGAUGUAGAGUUUTT | AAACUCUACAUCAUAUCCCTT |

**Supplemental Table 4. Cultured Cells**

| **Name** | **Source** | **Product number** |
| --- | --- | --- |
| Human umbilical vein endothelial cells | ATCC | Cat#PCS-100-013 |
| Human aortic endothelial cells | ATCC | Cat#PCS-100-011 |
| HEK293T | ATCC | Cat#CRL-11268 |
| THP-1 | Procell Life Science&Technology Co.,Ltd | Cat#CL-0233 |

**Supplemental Table 5. Ethical Approval Documentation.**

**
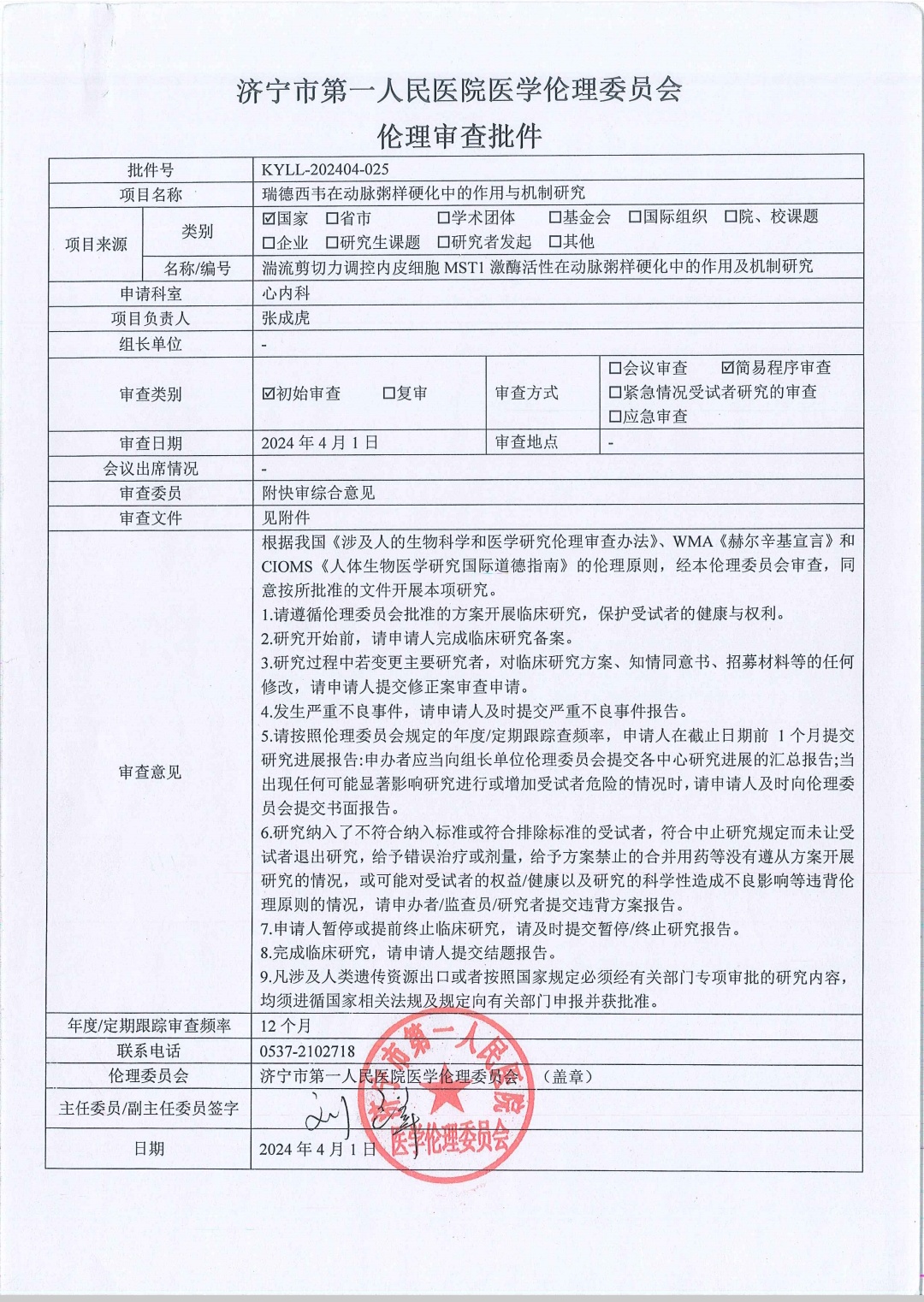
**
